# Complete microbial synthesis of crocetin and crocins from glycerol in *Escherichia coli*

**DOI:** 10.1186/s12934-023-02287-9

**Published:** 2024-01-04

**Authors:** Jun Ho Lee, Seong-Rae Lee, Sang Yup Lee, Pyung Cheon Lee

**Affiliations:** 1 Department of Molecular Science and Technology, Department of Applied Chemical and Biological Engineering, Ajou University, Woncheon-dong, Yeongtong-gu, Suwon, 16499 Republic of Korea; 2 Department of Chemical and Biomolecular Engineering, Korea Advanced Institute of Science and Technology (KAIST), 291 Daehak-ro, Yuseong-gu, Daejeon, 34141 Republic of Korea

**Keywords:** Crocetin, Crocin, Metabolic engineering, Saffron

## Abstract

**Background:**

Crocin, a glycosylated apocarotenoid pigment predominantly found in saffron, has garnered significant interest in the field of biotechnology for its bioactive properties. Traditional production of crocins and their aglycone, crocetin, typically involves extraction from crocin-producing plants. This study aimed to develop an alternative biosynthetic method for these compounds by engineering the metabolic pathways of zeaxanthin, crocetin, and crocin in *Escherichia coli* strains.

**Results:**

Employing a series of genetic modifications and the strategic overexpression of key enzymes, we successfully established a complete microbial pathway for synthesizing crocetin and four glycosylated derivatives of crocetin, utilizing glycerol as the primary carbon source. The overexpression of zeaxanthin cleavage dioxygenase and a novel variant of crocetin dialdehyde dehydrogenase resulted in a notable yield of crocetin (34.77 ± 1.03 mg/L). Further optimization involved the overexpression of new types of crocetin and crocin-2 glycosyltransferases, facilitating the production of crocin-1 (6.29 ± 0.19 mg/L), crocin-2 (5.29 ± 0.24 mg/L), crocin-3 (1.48 ± 0.10 mg/L), and crocin-4 (2.72 ± 0.13 mg/L).

**Conclusions:**

This investigation introduces a pioneering and integrated microbial synthesis method for generating crocin and its derivatives, employing glycerol as a sustainable carbon feedstock. The substantial yields achieved highlight the commercial potential of microbial-derived crocins as an eco-friendly alternative to plant extraction methods. The development of these microbial processes not only broadens the scope for crocin production but also suggests significant implications for the exploitation of bioengineered compounds in pharmaceutical and food industries.

**Supplementary Information:**

The online version contains supplementary material available at 10.1186/s12934-023-02287-9.

## Background

Crocin is an apocarotenoid digentiobiosyl ester of crocetin naturally found in saffron, primarily obtained from the red stigma of the *Crocus sativus* plant. The pharmacological benefits of crocin and its precursor crocetin, the aglycone of crocin, have been extensively documented in humans [1–4]. Currently, the organic solvent extract of the stigma is the primary source of crocetin and crocins. Per kilogram of dry saffron, this process typically requires between 110,000 and 170,000 flowers [5, 6]. However, producing crocin from saffron is also substantially influenced by environmental factors, such as light irradiation and temperature during the drying and extraction steps [7], as well as seasonal influences during cultivation. Because of these constraints, attempts at chemical synthesis have been reported, but no efficient alternative chemical method for producing crocin has been developed owing to poor stereospecificity and low efficiency [8, 9].

**Fig. 1:**
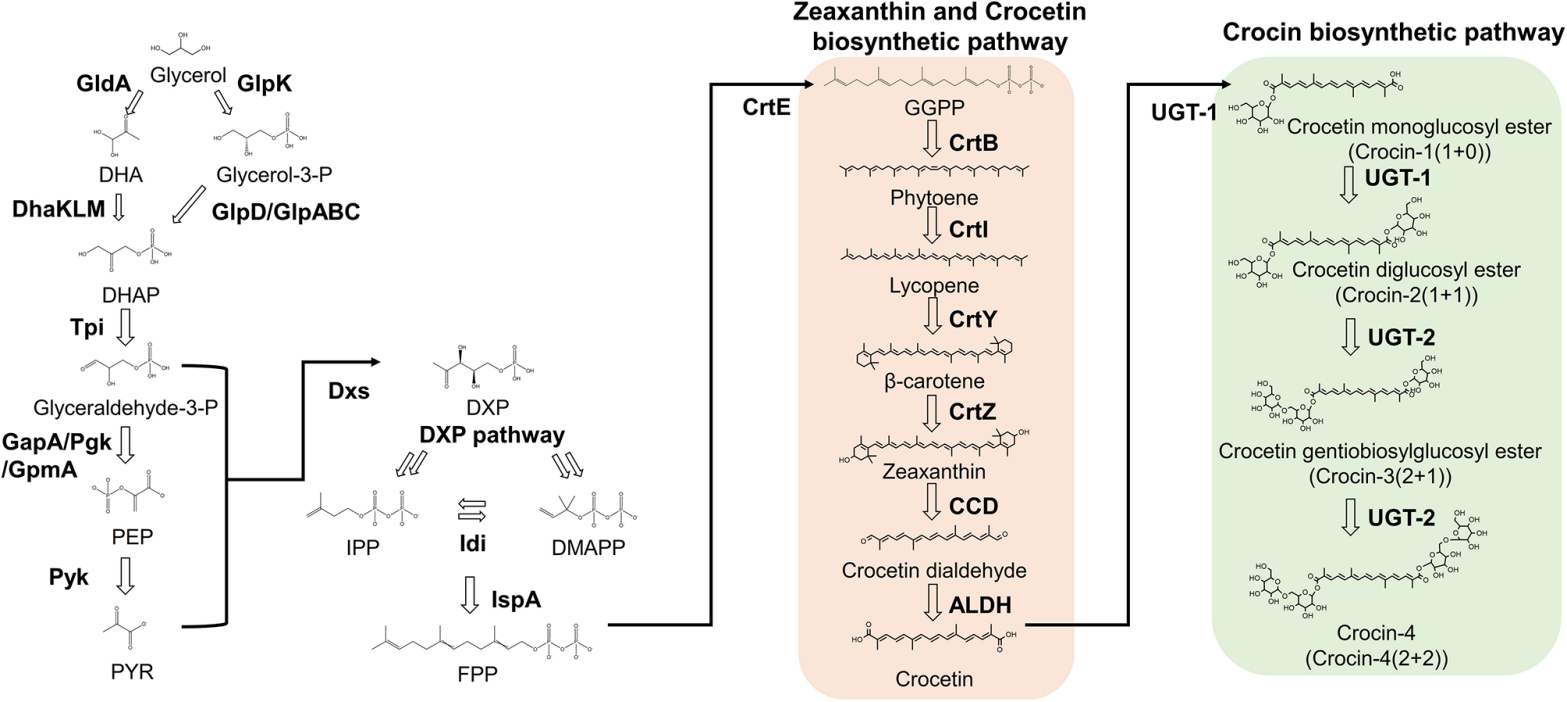
Engineered crocin biosynthetic pathway in *Escherichia coli*

A series of enzymatic reactions is necessary to synthesize crocetin and crocin from renewable carbon sources in microorganisms (Fig. 1). This includes the biosynthesis of β-carotene from farnesyl pyrophosphate (FPP) through sequential reactions catalyzed by geranylgeranyl diphosphate synthase (CrtE), phytoene synthase (CrtB), phytoene desaturase (CrtI), and lycopene cyclase (CrtY). Thereafter, β-carotene is hydroxylated to zeaxanthin by β-carotene hydroxylase (CrtZ), zeaxanthin is cleaved to crocetin dialdehyde by zeaxanthin cleavage dioxygenase (CCD), crocetin dialdehyde is oxidized to crocetin by crocetin dialdehyde dehydrogenase (crALDH), crocetin is first glycosylated to crocin mono/diglucosyl ester (crocin-1 and crocin-2) by uridine diphosphate (UDP)-glucuronosyltransferase (UGT-1), and finally crocin diglucosyl ester is glycosylated to crocin mono- or di-gentiobiosyl ester (crocin-3 and crocin-4) by UDP-glucuronosyltransferase (UGT-2) [10].

Independent research has investigated the different enzyme reaction steps in the crocin pathway for engineering microbial hosts to produce crocetin and crocin. For instance, researchers have focused on engineering the biosynthetic pathways of FPP and zeaxanthin, which are precursors for crocin [11, 12]. Recent discoveries in microbial genome data have unveiled new gene pathways, significantly advancing our understanding of zeaxanthin production in non-carotenogenic microorganisms [13–15]. A critical step in crocin biosynthesis is the symmetrical cleavage of zeaxanthin’s two 3-OH-β-ionone rings to yield crocetin dialdehyde. Researchers have identified and functionally expressed carotenoid cleavage dioxygenases (CCDs) from diverse sources. This progress addresses a key challenge in developing microbial pathways for crocin synthesis [6, 16, 17]. Similarly, genes encoding crALDH with high activity on crocetin dialdehyde have been thoroughly investigated, suggesting their importance in constructing the microbial crocin pathway [18–21]. Furthermore, gene mining and functional studies of two different types of UGT-1 and UGT-2 in microbial hosts [18, 19, 22, 23] have driven the metabolic engineering of total crocin biosynthesis in microorganisms. Consequently, through effectively redesigning and combining crocin pathway enzymes derived from endogenous and/or heterogeneous sources, it is possible to achieve a total microbial synthesis of crocin and its precursor crocetin using renewable carbon sources as an alternative method to plant extraction and chemical synthesis.

Crocetin and crocin can be synthesized using microbial processes employing microorganisms, including *Escherichia coli* and *Saccharomyces cerevisiae* [24–26]. Crocetin and crocin production via microbial processes can be divided into two approaches: total microbial synthesis of crocetin and crocin from a raw carbon source, such as glucose, and whole-cell (or enzymatic) biotransformation of crocetin into crocin. However, comprehensive engineering of the crocin pathway in microbial hosts, particularly for crocin-3 and crocin-4, has not been documented yet.

In this study, we successfully engineered *E. coli* strains for the simultaneous biosynthesis of microbial crocetin dialdehyde, crocetin, and all four types of crocins (crocin-1 to -4) from glycerol, employed as the carbon source. This was achieved by integrating novel variants of crALDH, UGT-1, and UGT-2 into the strains. Glycerol, known for its efficacy as a carbon source in carotenoid-producing recombinant microorganisms due to its ability to enhance production yields and reduce by-product formation [27, 28], was specifically chosen for its potential to optimize crocetin and crocin biosynthesis in the engineered *E. coli* strains.

Enzymes in the reconstructed crocin biosynthetic pathway are GldA: glycerol dehydrogenase; GlpK: glycerol kinase; DhaK: dihydroxyacetone kinase subunit K; DhaL: dihydroxyacetone kinase subunit L; DhaM: dihydroxyacetone kinase; GlpD: aerobic glycerol 3-phosphate dehydrogenase; GlpA: anaerobic glycerol-3-phosphate dehydrogenase subunit A; GlpB: anaerobic glycerol-3-phosphate dehydrogenase subunit B; GlpC: anaerobic glycerol-3-phosphate dehydrogenase subunit C; Tpi: triose-phosphate isomerase; GapA: glyceraldehyde-3-phosphate dehydrogenase; Pgk: phosphoglycerate kinase; GpmA: 2,3-bisphosphoglycerate-dependent phosphoglycerate mutase; Pyk: pyruvate kinase; Dxs: 1-deoxy-D-xylulose-5-phosphate synthase; Idi: isopentenyl diphosphate isomerase; IspA: farnesyl diphosphate synthase; CrtE: geranylgeranyl diphosphate synthase, CrtB: phytoene synthase, CrtI: phytoene desaturase; CrtY: lycopene cyclase; CrtZ: β-carotene hydroxylase; CCD: zeaxanthin cleavage dioxygenase; ALDH: crocetin dialdehyde dehydrogenase; UGT-1: crocetin glycosyltransferase; and UGT-2: diglycosylated crocin (crocin-2) glycosyltransferase.

## Results and discussion

### Construction of a zeaxanthin-producing *E. Coli* strain and determining temperature conditions for maximum yield

We designed five genes encoding zeaxanthin pathway enzymes (CrtE, CrtB, CrtI, CrtY, and CrtZ) [26] to be modularly expressed on the genome of an IPP-overproducing *E. coli* strain [29] to provide zeaxanthin as a substrate for the CCD enzyme in crocin biosynthesis (Fig. 1). As a result, the ZEA-1 strain was created (Additional file 1: Fig. S1A). We investigated four temperature control modes (constant maintenance at 20 °C, 30 °C, and 37 °C, as well as a shifting mode from 30 °C to 20 °C) in flask-scale cultivation, as prior research has shown that the production of carotenoids, including zeaxanthin, can be influenced by culture temperatures [30]. The ZEA-1 strain grew faster at higher temperatures, with the maximum optical density (OD_600_) value of approximately 11 at 30 ℃ and 37 ℃, an OD_600_ of 8 at 30 → 20℃, and an OD_600_ of 6 at 20℃ (Additional file 1: Fig. S1B). In contrast, zeaxanthin production tended to increase with lower culture temperatures. The highest amount of zeaxanthin (14.13 ± 1.55 mg/L) was obtained at 30 → 20 ℃, followed by 12.62 ± 1.26 mg/L at 20 ℃, 6.69 ± 1.00 mg/L at 30 ℃, and 2.32 ± 0.37 mg/L at 37 ℃ (Additional file 1: Fig. S1C). Based on these results, we selected a culture temperature of 30 → 20 ℃ or a fixed temperature of 20 ℃ for cultivating the ZEA-1 strain to supply zeaxanthin to CCD2 as a substrate in vivo.

### Construction of a crocetin dialdehyde-producing *E. Coli* strain and determining temperature conditions for maximum yield

**Fig. 2:**
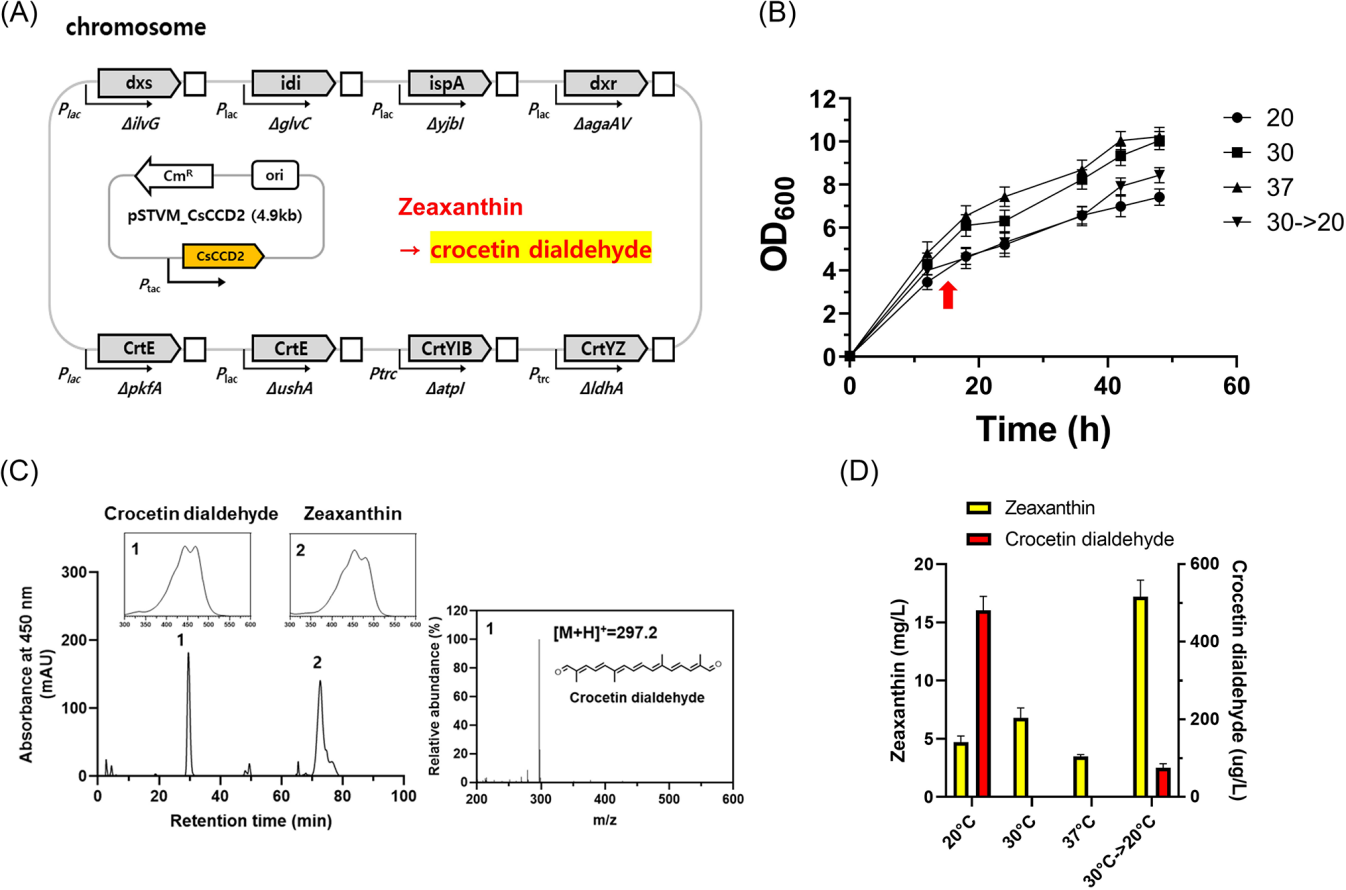
Schematic representation of the crocetin dialdehyde-producing Z1pC strain and the effect of culture temperatures on the growth and crocetin dialdehyde production in flask fermentations. **(A)** Schematic representation of crocetin dialdehyde-producing Z1pC strain construction via the codon-optimized synthetic CsCCD2 gene expression on a plasmid in the zeaxanthin-producing ZEA-1. **(B)** Cell growth of the Z1pC strain was grown in flasks and monitored at four culture temperatures. An arrow indicates the time of shifting from 30 to 20 ℃. **(C)** HPLC, UV/Vis, and LC-MS analysis of the extract of the Z1pC strains grown at 20 ℃. In the HPLC chromatogram, peak 1 corresponds to crocetin dialdehyde and peak 2, zeaxanthin. **(D)** Quantification of crocetin dialdehyde and zeaxanthin in the Z1pC strains grown at four different temperatures. All experiments were performed in biological triplicate, and error bars represent mean ± standard deviation (SD)

**Fig. 3:**
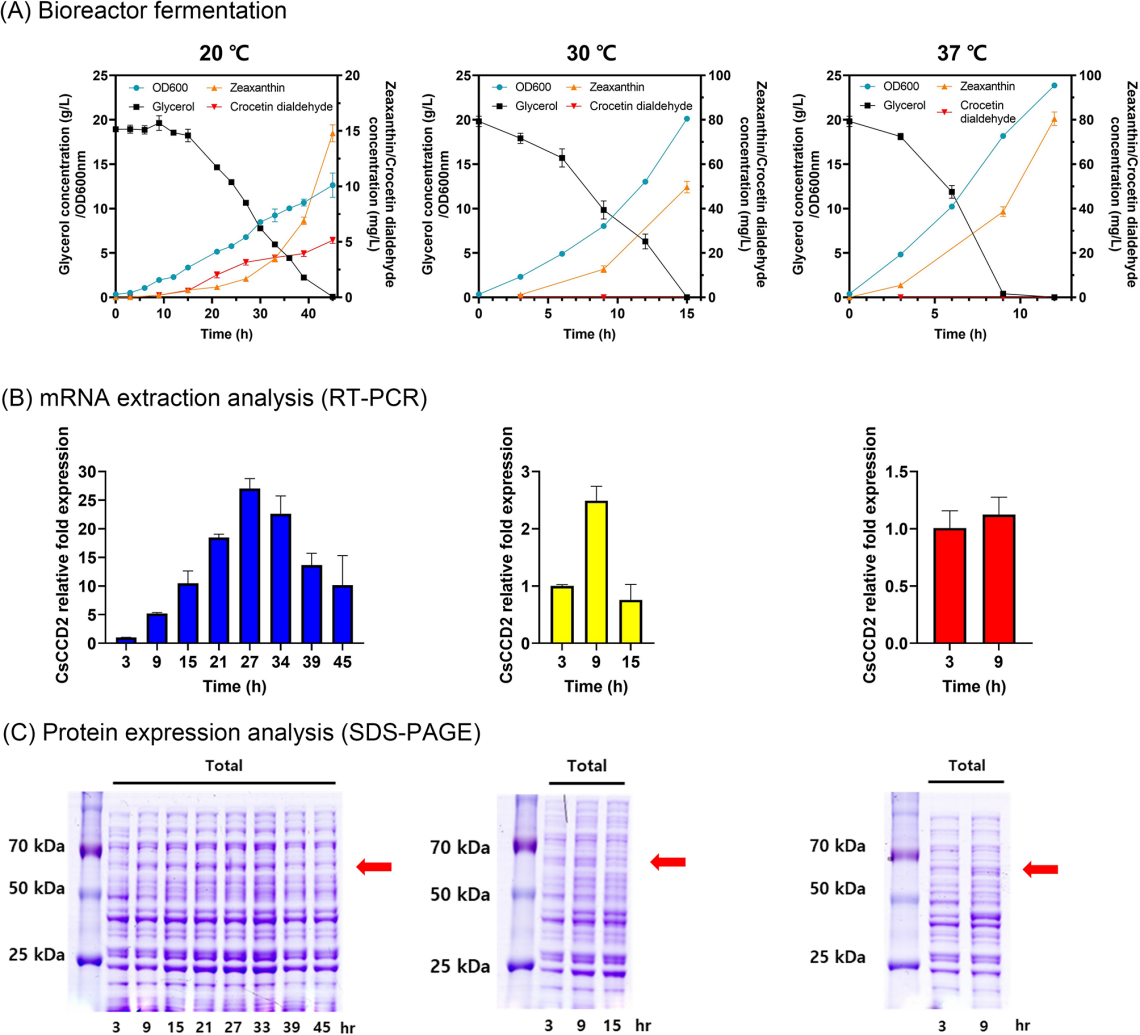
Effect of culture temperatures on the crocetin dialdehyde production of the Z1pC strain in batch bioreactor fermentations. Batch bioreactor fermentations of the crocetin dialdehyde-producing Z1pC strain were performed with the same culture parameters except for the culture temperature: 37 ℃, 30 ℃, or 20 ℃. **(A)** Cell growth, glycerol consumption, and crocetin dialdehyde production at three different temperatures. **(B)** The time-course mRNA expression level of CsCCD2 in Z1pC strain cultures at three different temperatures and analyzed using RT-qPCR. Relative expression level is presented by normalizing with the expression level of the *cys*G gene (a reference gene). **(C)** The time-course protein expression level of the CsCCD2 enzyme at three different temperatures was analyzed on the SDS-PAGE gels. An arrow indicates a band corresponding to the CsCCD2 enzyme of the calculated size of 63 kDa. All experiments, including bioreactor fermentations, were performed in biological triplicate, and error bars represent mean ± SD

Among CCD enzymes, which cleave the double bonds at the C7, C8, and C7’, C8’ positions of zeaxanthin [6], we selected the CsCCD2 enzyme from Crocus sativus. This enzyme is recognized for its cleavage activity in microorganisms [10, 18, 19, 31–33] and was utilized for constructing the crocetin dialdehyde pathway in the ZEA-1 strain. The codon-optimized synthetic CsCCD2 gene was designed to be expressed on the plasmid (as pSTVM_CsCCD2) in the ZEA-1 strain, generating the Z1pC strain to construct a crocetin dialdehyde pathway (Fig. 2A). Given that the functional expression of many plant-derived proteins in microbial hosts, including *E. coli*, can be significantly affected by culture temperatures, the effect of culture temperature on the growth and crocetin dialdehyde production of the Z1pC strain was investigated in flask-scale cultivation [10]. The highest growth (OD_600_ of 11) was observed at 30 ℃ and 37 ℃, followed by an OD_600_ of 9 at 30 → 20 ℃ and an OD_600_ of 8 at 20 ℃ (Fig. 2B). Notably, crocetin dialdehyde (λ_max_ = 447, [M-H]^−^ = 295.18, peak 1 in Fig. 2B) was detected alongside a zeaxanthin peak (peak 2 in Fig. 2C) in the Z1pC strain grown at both 20 °C and 30 → 20 ℃, indicating that CsCCD2 was functionally active at only 20 °C and 30 → 20 ℃ but not at 30 ℃ and 37 ℃. Crocetin dialdehyde concentrations were determined to be 0.48 ± 0.03 mg/L at 20 ℃ and 0.07 ± 0.01 mg/L at 30 → 20 ℃ (Fig. 2D). The considerable accumulation of zeaxanthin in the Z1pC strain (17.2 ± 1.4 mg/L at 30 → 20 ℃ and 4.7 ± 0.53 mg/L at 20 °C) shows limited cleavage of zeaxanthin into crocetin dialdehyde, possibly due to the weak activity or low expression of CsCCD2. As a result, the batch bioreactor fermentation of the Z1pC strain at different temperatures of 20, 30, or 37 °C was used to evaluate the kinetics of crocetin dialdehyde formation and the expression level of CsCCD2. The highest concentration of crocetin dialdehyde (5.14 ± 0.28 mg/L) was produced at 45 h in the Z1pC strain cultivated at 20 ℃ (Fig. 3A), while zeaxanthin rapidly accumulated after 27 h, reaching up to 14.5 ± 1.5 mg/L at 45 h. Crocetin dialdehyde formation was highly correlated with CsCCD2 transcription levels 20, 30, or 37 °C: the highest mRNA level of the CsCCD2 was 27-fold at 20 ℃, followed by 2.5-fold at 30 ℃, and 1.2-fold at 37 ℃ (Fig. 3B).

Notably, after reaching their peak at 20 ℃ (27 h at 20 ℃ in Fig. 3B), the mRNA levels of the CsCCD2 enzyme gradually declined. This decrease coincided with rapid zeaxanthin accumulation and a slower rate of crocetin dialdehyde synthesis (20 ℃ in Fig. 3A). CsCCD2 protein expression correlated with the mRNA level (Fig. 3C), demonstrating that CsCCD2 functional expression influenced crocetin dialdehyde synthesis in the Z1pC strain. The protein expression level of CsCCD2 is strongly and positively related to crocetin dialdehyde synthesis. As a result, altering CsCCD2 mRNA expression/stability could be a potential target for increasing crocetin dialdehyde synthesis [34].

Furthermore, crocetin dialdehyde may be directed towards an unknown degradation pathway, potentially involving the promiscuous activities of endogenous reductase(s) in *E. coli*, as has been observed in retinoids-producing *E. coli* strains [34, 35]. Hence, genome editing of the gene(s) that negatively influence crocetin dialdehyde formation and stability would be required to create crocetin dialdehyde successfully.

### Construction of a crocetin biosynthetic pathway by employing a crocetin dialdehyde dehydrogenase

**Fig. 4:**
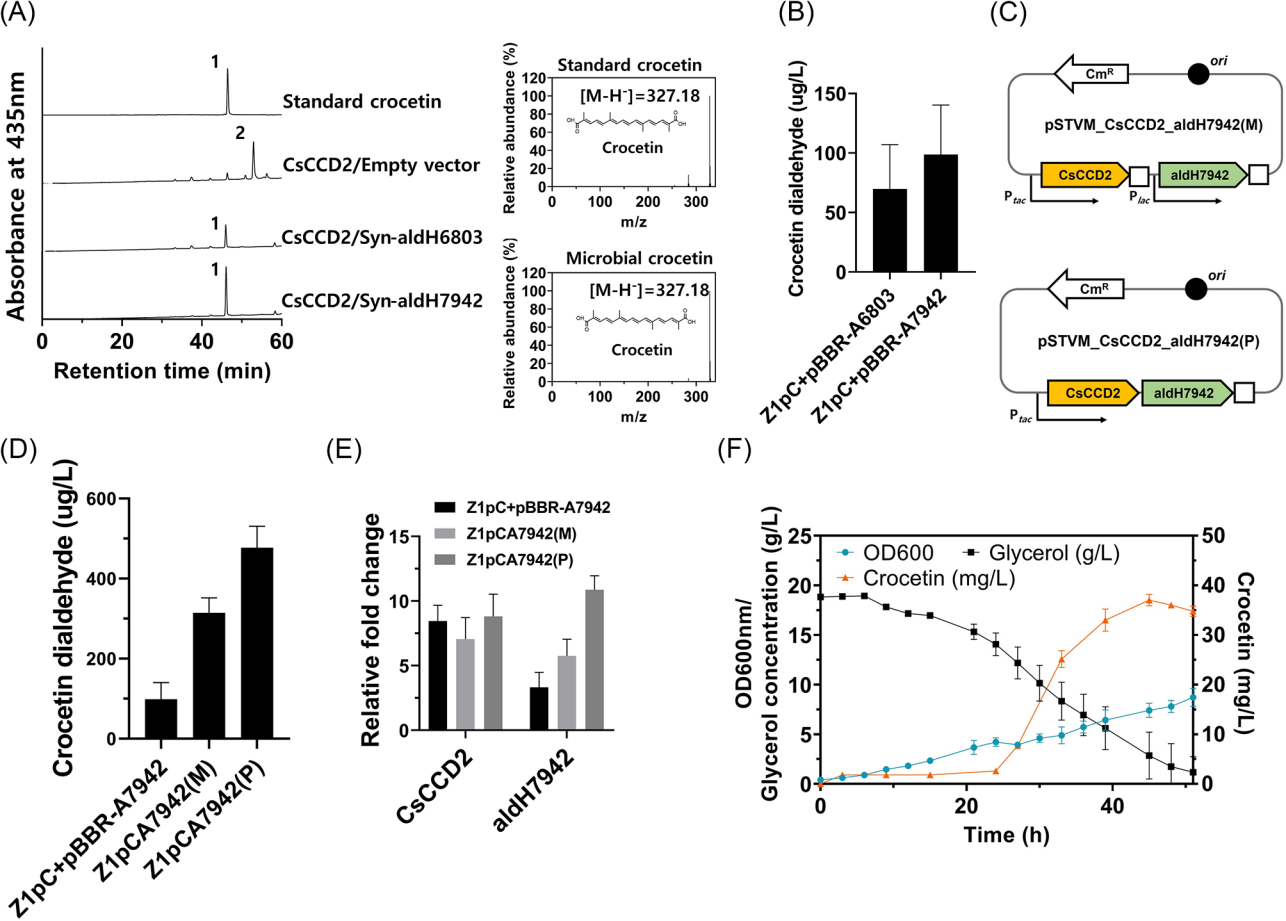
Construction of crocetin pathway and crocetin production in flask and batch bioreactor fermentations. **(A)** HPLC and LG-MS analysis of an extract of the Z1pC strain expressing ALDH6803 (as pBBR-A6803) from *Synechocystis sp*. PCC6803 or ALDH7942 (pBBR-A7942) from *S. elongates* PCC7942. In the HPLC chromatogram, peak 1 corresponds to crocetin, and peak 2, to crocetin dialdehyde. **(B)** Quantifying crocetin production in the Z1pC strain expressing ALDH6803 or ALDH7942 in flask cultures. **(C)** Schematical description of individual modular (upper) and polycistronic (lower) expression systems of CsCCD2 and ALDH7942. **(D)** Quantifying crocetin production in Z1pC strain expressing ALDH7942 via polycistronic or modular mode in flask cultures. **(E)** mRNA expression levels of CsCCD2 and ALDH7942 via two plasmid systems (noted as Z1pC + pBBR-A7942) and one plasmid system with polycistronic (noted as Z1pCA7942(P)) or individual modular expression (noted as Z1pCA7942(M)). Relative expression was presented by normalizing with the expression level of the *cys*G gene (a reference gene). **(F)** Batch bioreactor fermentations of the crocetin-producing Z1pCA7942(P) strain at 20 ℃. All experiments, including bioreactor fermentation, were done in biological triplicate, and error bars represent mean ± SD.

The reconstructed crocetin dialdehyde pathway was further modified to produce crocetin by introducing a crocetin dialdehyde dehydrogenase (crALDH) into the Z1pC strain. As gene expression from microbial sources is generally more suitable for heterologous expression in microbial strains, ALDH6803 of *Synechocystis* sp. PCC6803 [21] was chosen for the crocetin pathway engineering. Furthermore, an uncharacterized ALDH7942 of *Synechocystis elongatus* PCC7942 was selected as a candidate crALDH based on its homology to ALDH6803 and CsALDH31l [19] (Additional file 1: Fig. S2). Plasmids expressing ALDH6803 or ALDH7942 (named pBBR-A6803 and pBBR-A7942, respectively) were transformed into the Z1pC strain, yielding the Z1pCpA6803 and Z1pCpA7942 strains. Following cultivation at 20 °C in flasks, crocetin production of each strain was investigated. HPLC analysis revealed the presence of a small new peak (peak 1 in Fig. 4A) in the extracts of both Z1pCpA6803 and Z1pCpA7942 strains. LC-MS and UV/Vis analysis confirmed that This peak was identified as crocetin (λ_max_ = 425, [M-H]^−^ = 327.18), demonstrating that ALDH6803 and ALDH7942 were both capable of oxidizing crocetin dialdehyde to crocetin. Quantitative analysis showed that the Z1pCpA7942 strain produced 98.65 ± 41.65 µg/L of crocetin, whereas the Z1pCpA6803 strain produced 69.86 ± 37.25 µg/L (Fig. 4B). As ALDH7942 demonstrated higher activity in crocetin production than ALDH6803, ALDH7942 was selected for further optimization of the crocetin pathway using two different expression systems: (1) individual modular expression and (2) polycistronic expression. In the individual modular expression system, two genes encoding ALDH7942 and CsCCD2 were co-expressed as separate transcripts, each with its promoter and terminator (Z1pCA7942(M), Fig. 4C). In the polycistronic expression system, the two genes were co-expressed as a single transcript with one promoter and terminator (Z1pCA7942(P) Fig. 4C). Quantitative analysis revealed that the Z1pCA7942(P) strain produced 477.15 ± 53.49 µg/L of crocetin, while the Z1pCA7942(M) strain produced 314.29 ± 37.45 µg/L (Fig. 4D). Crocetin levels were 3.2-fold and 4.8-fold higher, respectively, than the crocetin production of 98.65 ± 41.65 µg/L in the Z1pCpA7942 strain. Transcriptional analysis of ALDH7942 indicated that there was a positive correlation between crocetin production and the mRNA expression level of ALDH7942 (Fig. 4E). ALDH7942 mRNA expression was highest in polycistronic expression (11-fold), followed by individual modular expression (6-fold), and two-plasmid expression (3-fold). Notably, the mRNA expression level of CsCCD2 remained relatively constant, ranging from 7-fold to 9-fold, compared to the significant changes in the mRNA expression level of ALDH7942. A batch bioreactor fermentation at 20 °C was conducted to acquire insights into the kinetics of crocetin synthesis in the Z1pCA7942(P) strain. The Z1pCA7942(P) strain exhibited robust growth, reaching an OD_600_ of 8.72, and produced up to 34.77 ± 1.03 mg/L of crocetin after 51 h of fermentation, without the accumulation of crocetin dialdehyde (Fig. 4F).

### Construction of a crocin-2 biosynthetic pathway using a crocetin glucosyltransferase

**Fig. 5:**
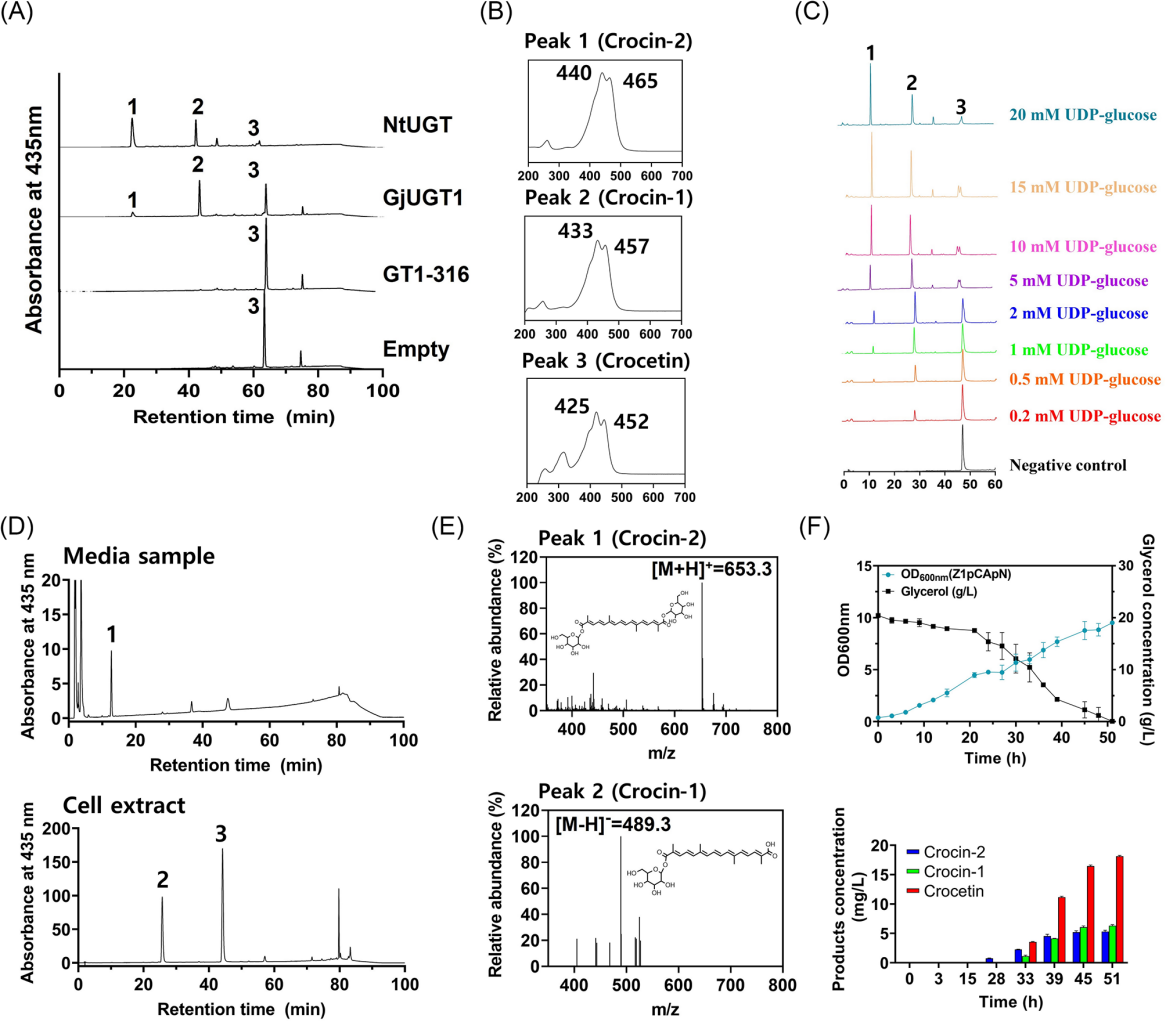
Construction of crocin-1 and 2 pathways and crocin-1 and 2 production in batch bioreactor fermentations. **(A)** HPLC and UV/Vis analysis of in vitro activity of three first-step UGT candidates (GjUGT1, GT1-316, and NtUGT) for determining the glycosylation activity on crocetin. Peak 1 corresponds to crocin-2; peak 2, crocin-1; peak 3, crocetin. **(B)** UV/VIS spectrum corresponds to each HPLC chromatogram peak (**A**). **(C)** HPLC analysis of UDP-glucose-dependent activity of NtUGT in the in vitro reaction. The assignment of peaks is the same as that of (**A**). **(D)** HPLC analysis of culture medium (upper) and Z1pCA7942(P) cell extract pN. The assignment of peaks in the HPCL chromatogram is the same as that of **(A)**. **(E)** LC/MS analysis of crocin-2 [peak 1 in (**D**)] and crocin-1[peak 2 in (**D**)]. **(F)** Cell growth and glycerol consumption in batch bioreactor fermentations of the Z1pCA7942(P)pN strain at 20℃ (upper). Quantifying the time-course production of crocin-1, crocin-2, and crocetin in bioreactor batch fermentations (lower). Bioreactor fermentations were performed in biological triplicate, and error bars represent mean ± SD

The construction of a crocin-2 biosynthetic pathway requires a crocetin glycosyltransferase (UGT) capable of glycosylating specific carboxyl groups of crocetin to produce one-glycosylated crocin-1 and two-glycosylated crocin-2 (Fig. 1). GjUGT1 (or UGT75L6) from *Gardenia jasminoides* was shown to have crocetin glycosylation activity [22] and was selected as a candidate UGT for constructing the crocin-2 pathway in the Z1pCA7942(P) strain. A homology search using the amino acid sequence of GjUGT1 yielded four UGT candidates (NtUGT, GT1-316, StUGT, and FaGT2) (Additional file 1: Fig. S2). The crocetin glycosylation activity of the five codon-optimized synthetic UGTs was tested in vitro before introducing them into the crocetin-producing Z1pCA7942(P) strain. StUGT and FaGT2 were not proteins expressed in *E. coli* BL21(DE3), three UGTs (GjUGT1, NtUGT, and GT1-316) in a crude protein extract were tested in vitro with crocetin and UDP-glucose as co-substrates. HPLC analysis of the assay mixtures revealed the presence of two new peaks (peaks 1 and 2 in Fig. 5A) in the extracts of both GjUGT1 and NtUGT. UV/Vis spectra analysis confirmed that these peaks exhibited UV/Vis spectra (Fig. 5B) similar to those of crocin-1 (λ_max_ = 433) and crocin-2 (λ_max_ = 440) (Demurtas et al., 2018). Peak areas of peaks 1 and 2 in the HPLC chromatogram increased with UDP-glucose concentration (Fig. 5C), indicating that both GjUGT1 and NtUGT had glycosylation activity on crocetin, resulting in one-glycosylated crocetin (crocin-1, peak 2) and two-glycosylated crocetin (crocin-2, peak 1). Based on a comparison of the peak area, which represented the activity of GjUGT1 and NtUGT, NtUGT was chosen as the first step UGT for constructing the crocin-2 pathway in the Z1pA7942(P) strain.

The NtUGT gene was engineered to be constitutively expressed on a plasmid in the Z1pA7942(P) strain, yielding the Z1pCA7942(P)pN strain. Analysis of the culture medium and cell crude extract of the Z1pCA7942(P)pN strain grown in flasks using HPLC and UV/Vis revealed the presence of a peak similar to crocin-2 in the culture medium (peak 1 in the upper panel of Fig. 5D) and a peak identical to crocin-1 in the cell extract (peak 2 in the lower panel of Fig. 5D). LC/MS and UV/Vis analysis confirmed that peak 1 corresponded to crocin-2 (λ_max_ = 440, [M-H]^−^ = 653.3) and peak 2 corresponded to crocin-1 (λ_max_ = 433, [M-H]^−^ = 489.3), confirming the functionality of NtUGT as a first step UGT capable of glycosylating crocetin in *E. coli*.

To gain insight into the production kinetics of crocin-1, crocin-2, and cell growth, batch bioreactor fermentation of the Z1pCA7942(P)pN strain was performed. The strain exhibited growth up to an OD_600_ of 9.51 with complete consumption of 20 g/L glycerol (Fig. 5F). Crocin-2 was detected after 25 h of culture and steadily increased to a concentration of 5.29 ± 0.24 mg/L at 51 h. Similarly, crocin-1 was detected at 33 h and gradually increased to a concentration of 6.29 ± 0.19 mg/L at 51 h. Notably, crocetin accumulation began at 33 h and rapidly increased to 18.12 ± 0.14 mg/L at 51 h. The declining production rate of crocin-1 and crocin-2, coupled with the increasing accumulation of crocetin, suggests that the glycosylation reaction of crocetin may be limited due to unknown physiological changes and/or metabolic flux imbalance, such as insufficient UDP-glucose supply.

### Construction of a crocin-4 biosynthetic pathway using a crocin-2 glucosyltransferase

**Fig. 6:**
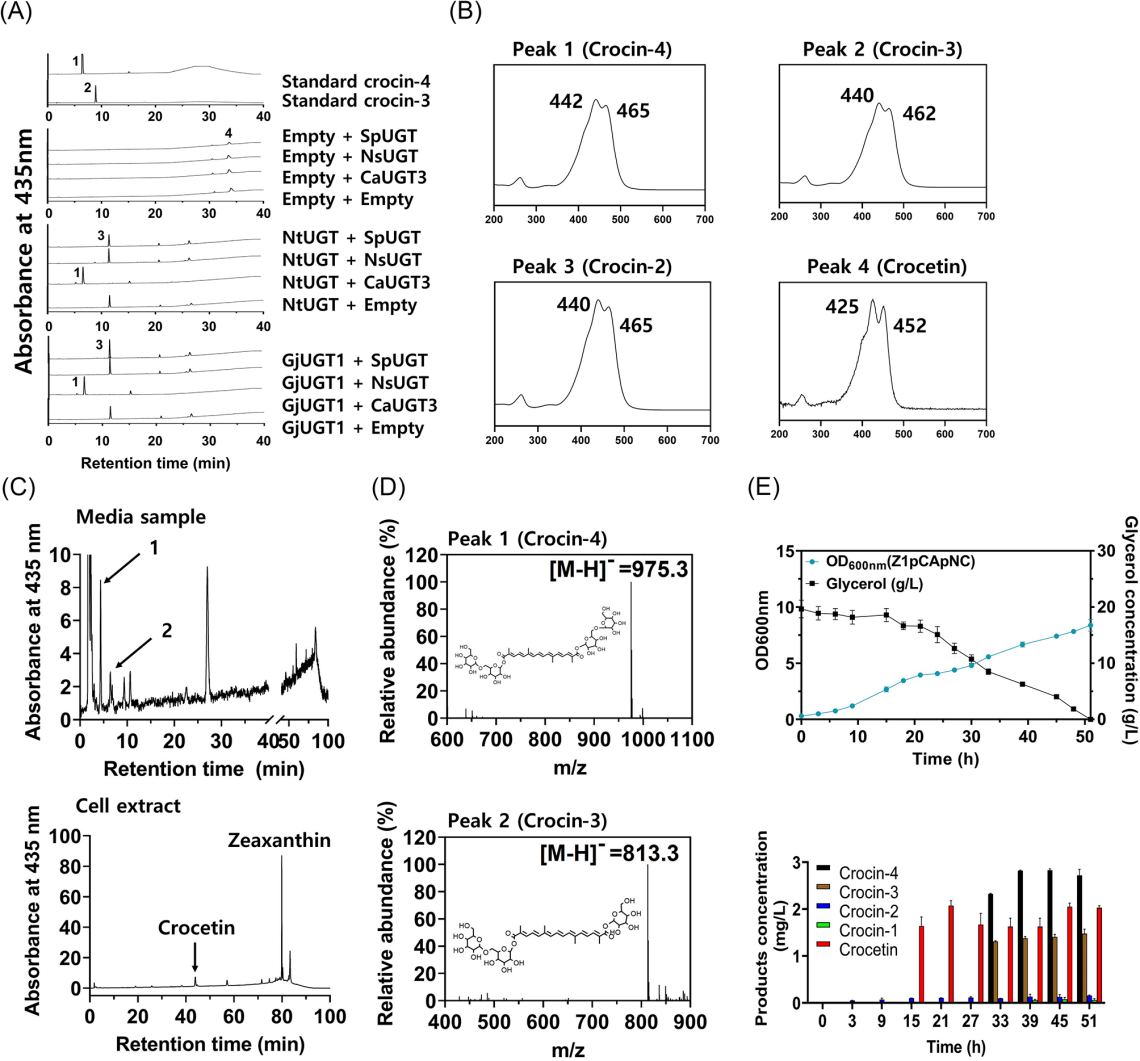
Construction of crocin-3/4 pathway and crocin-3/4 production in batch bioreactor fermentations. **(A)** HPLC and UV/Vis analysis of in vitro activity of three second step UGT candidates (GjUGT1, GT1-316, and NtUGT) for determining the glycosylation activity on crocin-1/2. In vitro reaction was performed with combinations of the first reaction of two first-step UGTs (GjUGT1 and NtUGT), which converted crocetin into crocin-1/2, and the second reaction of three second-step UGT (SpUGT, NsUGT, and CaUGT3): (upper) crocin-3/4 standard (upper-middle) empty + SpUGT, empty + NsUGT, empty + CaUGT3, empty + empty (a control); (lower-middle) NtUGT + SpUGT, NtUGT + NsUGT, NtUGT + CaUGT3, NtUGT + empty (a control); (lower) GjUGT1 + SpUGT, GjUGT1 + NsUGT, GjUGT1 + CaUGT3, GjUGT1 + empty (a control). Peak 1 corresponds to crocin-4; peak 2, crocin-2; peak 3, crocetin. **(B)** UV/VIS spectrum corresponds to each HPLC chromatogram peak **(A)**. **(C)** HPLC analysis of culture medium (upper) and Z1pCA7942(P)pNC cell extract. **(D)** LC-MS analysis of crocin-4 and crocin-3. **(E)** Cell growth and glycerol consumption in bioreactor batch fermentations of the Z1pCA7942(P)pNC (upper). The time-course production of crocin-1/2/3/4 in bioreactor batch fermentations of the Z1pCA7942(P)pNC (lower). Bioreactor fermentations were performed in biological triplicate, and error bars represent mean ± SD

In a previous study on crocin biosynthesis [22], the UGT enzyme GjUGT9 (or UGT94E5) was found to have glycosylation activity on the glucose moiety of crocin-2 in a plant, *G. jasminoides*, resulting in the formation of crocetin digentiobiosyl-ester (crocin-4, in Fig. 1). Based on amino acid homology with the query amino acid sequence of GjUGT9 and phylogenetic relatedness (Additional file 1: Fig. S3), three UGT candidates (SpUGT, NsUGT, and CaUGT3) were identified as potential second step UGTs in the construction of the crocin-4 biosynthetic pathway in the crocin-2 producing Z1pCA7942(P)pN strain. To investigate the in vitro activity of the second step UGTs, synthetic proteins of SpUGT, NsUGT, and CaUGT3 were expressed in a crude protein extract and subjected to an in vitro assay. UDP-glucose and a first-step UGT reaction mixture (GjUGT1 or NtUGT) were provided as substrates for the second step UGTs. HPLC and UV/Vis analysis of the assay mixtures revealed the presence of a highly polar peak (peak 1 in Fig. 6A) with a UV/Vis spectrum similar to that of crocin-4 (λ_max_ = 442) (Fig. 6B) only in the reaction mixtures containing CaUGT3 (i.e., CaUGT3 + GjUGT1 and CaUGT3 + NtUGT). This demonstrated that CaUGT3 could glycosylate crocin-2, whereas SpUGT and NsUGT did not. Based on these findings, CaUGT3 was chosen as the second-step UGT for constructing the crocin-4 pathway in the Z1pCA7942(P)pN strain.

The CaUGT3 gene in the Z1pCA7942(P) strain was modified to be co-expressed with NtUGT in a polycistronic module on a plasmid, resulting in the Z1pCA7942(P)pNC strain. HPLC and UV/Vis spectroscopy of the culture medium and cell extract from the Z1pCA7942(P)pNC strain cultured in flasks indicated the existence of two novel peaks (peaks 1 and 2 in Fig. 6C) with UV/Vis spectra similar to those of crocin-4 and crocin-3 and exclusively in the culture medium. Crocetin and zeaxanthin were detected in the cell extracts. LC/MS analysis (Fig. 6D) verified that peak 1 corresponded to crocin-4 (λ_max_ = 442, [M-H]^−^ = 975.3) and peak 2 to crocin-3 (λ_max_ = 440, [M-H]^−^ = 813.3), indicating that CaUGT3 functioned as a second step UGT capable of glycosylating crocin-2.

The Z1pCA7942(P)pNC strain achieved an OD_600_ of 8.38 after 51 h of batch bioreactor fermentation, with complete consumption of 20 g/L glycerol (Fig. 6E). Crocin-4 production began at 31 h and peaked at 2.72 ± 0.13 mg/L at 45 h, remaining relatively constant until 51 h. Similarly, crocin-3 production began at 33 h and increased to 1.48 ± 0.10 mg/L after 51 h. Crocetin began to accumulate at 15 h and remained at a concentration of 2.03 ± 0.04 mg/L, while crocin-1 and crocin-2 did not appreciably accumulate (0.16 ± 0.01 mg/L of crocin-2, 0.06 ± 0.03 mg/L of crocin-1; lower panel in Fig. 6E).

## Conclusion

As saffron pigments such as crocetin and crocin have gained increasing interest in the biotechnological and pharmaceutical industries, many microbial techniques, including biotransformation, have recently been used for their synthesis (Table 1). However, the reported microbial processes did not achieve the desired total synthesis of crocin-3 and crocin-4, the primary pigments found in plants, using renewable resources such as glycerol. By culturing metabolically engineered *E. coli* strains on glycerol, this study provides the first effective microbial total synthesis of four types of crocins (crocin-1, crocin-2, crocin-3, and crocin-4), as well as crocetin. Further optimization of the microbial processes for crocin production can be pursued to achieve higher yields of crocetin and crocin. Consequently, these crocetin- and crocin-producing strains serve as a valuable platform for commercializing microbial crocins and discovering new enzymes involved in crocin biosynthesis.

**Table 1:** Microbial crocetin/crocin production

Production host	Type of product	Method of Synthesis	Carbon source	Titers/ Yields (g-product/g-carbon source) *	Culture type	References
*S. cerevisiae*	Crocetin	Total synthesis	Glucose	6.28 mg/L / 0.012%	Fed-batch bioreactor	[10]
*S. cerevisiae*	Crocetin	Total synthesis	Glucose	12.43 ± 0.62 mg/L / 0.017%	Fed-batch bioreactor	[36]
*S. cerevisiae*	Crocetin	Total synthesis	Glucose, Galactose	139.67 ± 2.24 µg/g-DCW** / - ***	Shake-flask	[37]
*E. coli*	Crocetin	Total synthesis	Glycerol	4.42 mg/L / 0.029%	Shake-flask	[38]
*E. coli*	Crocin-1	Total synthesis	Glycerol	-	Shake-flask	[38]
*E. coli*	Crocin-4	Microbial biotransformation	Glucose	12.5 mg/L / 0.025%	Shake-flask	[18]
*E. coli*	Crocin-1, Crocin-2	Enzymatic Biotransformation	-	476.8 mg/L	NA****	[8]
*E. coli*	Crocetin	Total synthesis	Glycerol	34.77 ± 1.03 mg/L / 0.173%	Batch bioreactor	This study
*E. coli*	Crocin-1	Total synthesis	Glycerol	6.29 ± 0.19 mg/L / 0.031%	Batch bioreactor	This study
*E. coli*	Crocin-2	Total synthesis	Glycerol	5.29 ± 0.24 mg/L / 0.026%	Batch bioreactor	This study
*E. coli*	Crocin-3	Total synthesis	Glycerol	1.48 ± 0.10 mg/L / 0.007%	Batch bioreactor	This study
*E. coli*	Crocin-4	Total synthesis	Glycerol	2.72 ± 0.13 mg/L / 0.013%	Batch bioreactor	This study

* Yields were either calculated or adopted based on data derived from both published references and our study

** ‘DCW’ means ‘Dried Cell Weight’

*** ‘-’ represents ‘No Data Available’

**** ‘’’NA’ indicates ‘Not Applicable’

## Materials and methods

### Strains, media, and culture conditions

All strains used in this study are listed in Table 2. The *E. coli* MG1655 strain served as the foundation for engineering the zeaxanthin and crocin pathways, while *E. coli* TOP10 was used for gene cloning and *E. coli* BL21(DE3) was used for protein expression. *E. coli* was cultured aerobically at 30℃, 37℃, or 42℃ in Luria broth (LB) containing Tryptone (10 g/L), Yeast extract (5 g/L), and NaCl (5 g/L) during strain construction. A single colony grown on an LB-agar plate with or without antibiotics (100 µg/ml ampicillin, 50 µg/ml chloramphenicol, or 30 µg/ml kanamycin) was inoculated into culture tubes containing 4 mL of LB, with or without antibiotics, and cultured overnight at 37℃ and 250 rpm to produce zeaxanthin and crocin. Cell growth was assessed by measuring the optical density at a wavelength of 600 nm (OD_600_) using a SpectraMax Plus384 spectrophotometer (Molecular Devices, San Jose, CA, USA).

**Table 2:** *E. coli* strains and plasmids used in this study

Strain/plasmid	Relevant property		Source
**strain**			
MG1655	F−, λ−, rph-1		KCTC
MGI	MG1655 ilvGΔ::PLac-dxs, glvCΔ::PLac-idi, yjbIΔ::PLac-ispA, agaAVΔ::PLac-dxr		[39]
MGI2E	MGI (pfkAΔ::PLac-CrtE, ushAΔ::PLac-CrtE)		This study
ZEA-1	MGI2E (atpIΔ::Ptrc-CrtYIB, ldhAΔ::Ptrc-YZ)		This study
Z1pC	ZEA-1 harboring pSTVM_CsCCD2		This study
Z1pCpA6803	ZEA-1 harboring pSTVM_CsCCD2 and pBBR-A6803		This study
Z1pCpA7942	ZEA-1 harboring pSTVM_CsCCD2 and pBBR-A7942		This study
Z1pCA7942(M)	ZEA-1 harboring pSTVM_CsCCD2_Syn-aldH7942(M)		This study
Z1pCA7942(P)	ZEA-1 harboring pSTVM_CsCCD2_Syn-aldH7942(P)		This study
Z1pCA7942(P)pN	ZEA-1 harboring pSTVM_CsCCD2_Syn-aldH7942(P) and pUCrop_NtUGT		This study
Z1pCA7942(P)pNC	ZEA-1 harboring pSTVM_CsCCD2_Syn-aldH7942(P) and pUCrop_NtUGT_CaUGT3		This study
TOP10	F-mcrAΔ(mrr-hsdRMS-mcrBC) φ80lacZΔM15 ΔlacX74 nupG recA1 araD139 Δ(ara-leu)7697 galE15 galK16 rpsL(StrR) endA1		Invitrogen
BL21 (DE3)	F^–^*ompT gal dcm lon hsdSB*(*r*_*B*_^–^*m*_*B*_^–^) λ(DE3 [*lacI lacUV5*-*T7p07 ind1 sam7 nin5*]) [*malB*^+^]_K−12_(λ^S^)		NEB
*Synechocystis sp.* PCC 6803	wild type strain of *Synechocystis sp*.PCC6803		
*Synechocystis elongatus* PCC 7942	wild type strain of *S. elongatus* PCC7942		
**Plasmid**			
pKK223-3	Amp^r^, *tac* promoter, pBR322 ori, rop		Pharmacia
pTrc99A	Amp^r^, *trc* promoter, pBR322 ori		[32]
pUCM	Amp^r^, constitutively expressed *lac* promoter, pUC ori		[29]
pUCrop	Amp^r^, constitutively expressed *lac* promoter, pUC ori, rop		[39]
pET21a(+)	Amp^r^, T7 promoter, C-terminal His-tag sequence, f1 ori, rop,		Novagen
pSTVM	Cm^r^, Cloning/expression vector removing *lac* promoter from pSTV29		[33]
pBBR1MCS-2	Km^r^, Cloning vector		[40]
pUCM_CrtE	Amp^r^, pUCM derivative, expression vector for *crtE* from *P. ananatis*		[26]
pTrc99A_YIB	Amp^r^, pTrc99A derivative, expression vector for *crtYIB* from *P. ananatis crtYIB* operon at *EcoRI/HindIII* site		This study
pKK_CsCCD2	Amp^r^, pKK-223-3 derivative, expression vector for *CsCCD2* from *C.sativus* at *EcoRI/HindIII site*		This study
pSTVM_CsCCD2	Cm^r^, pSTVM derivative, expression vector for *CsCCD2* from *C.sativus* under *tac* promoter at *BglII/NotI site*		This study
pUCM_Syn-aldH6803	Amp^r^, pUCM derivative, expression vector for *aldH* from *Synechocystis* sp. PCC6803 (Syn_aldH6803) at *XbaI/EcoRI site*		This study
pUCM_Syn-aldH7942	Amp^r^, pUCM derivative, expression vector for *aldH* from *Synechocystis* sp. PCC7942 (Syn_aldH7942) at *XbaI/EcoRI site*		This study
pBBR-A6803	Km^r^, pBBR1MCS-2 derivative, expression vector for *aldH* from *Synechocystis* sp. PCC6803 (Syn_aldH6803) under *lac* promoter		This study
pBBR-A7942	Km^r^, pBBR1MCS-2 derivative, expression vector for *aldH* from *S. elongatus* PCC7942 (Syn_aldH7942) under *lac* promoter		This study
pSTVM_CsCCD2_Syn-aldH6803(M)	Cm^r^, pSTVM derivative, individual modular expression vector for *CsCCD2* and Syn_aldH6803		This study
pSTVM_CsCCD2_Syn-aldH7942(M)	Cm^r^, pSTVM derivative, individual modular expression vector for *CsCCD2* and Syn_aldH7942		This study
pSTVM_CsCCD2_Syn-aldH6803(P)	Cm^r^, pSTVM derivative, polycistronic expression vector for *CsCCD2* and Syn_aldH6803		This study
pSTVM_CsCCD2_Syn-aldH7942(P)	Cm^r^, pSTVM derivative, polycistronic expression vector for *CsCCD2* and Syn_aldH7942		This study
pUCrop_NtUGT	Amp^r^, pUCrop derivative, expression vector for NtUGT at *XbaI/XmaI site*		This study
pUCrop_NtUGT_CaUGT3	Amp^r^, pUCrop derivative, expression vector for NtUGT and CaUGT3 at *XbaI/NotI site*		This study
pET21a(+)_GjUGT1	Amp^r^, pET21a(+) derivative, inducible expression of 6xHis-tagged GjUGT gene at *EcoRI/XhoI site*		This study
pET21a(+)_GT1-316	Amp^r^, pET21a(+) derivative, inducible expression of 6xHis-tagged GT1-316 gene at *BamHI/SalI site*		This study
pET21a(+)_NtUGT	Amp^r^, pET21a(+) derivative, inducible expression of 6xHis-tagged NtUGT gene at *BamHI/HindIII site*		This study
pET21a(+)_FaGT2	Amp^r^, pET21a(+) derivative, inducible expression of 6xHis-tagged FaGT2 gene at *BamHI/HindIII site*		This study
pET21a(+)_StUGT	Amp^r^, pET21a(+) derivative, inducible expression of 6xHis-tagged StUGT gene at *EcoRI/XhoI site*		This study
pET21a(+)_CaUGT3	Amp^r^, pET21a(+) derivative, inducible expression of 6xHis-tagged CaUGT3 gene at *BamHI/SalI site*		This study
pET21a(+)_NsUGT	Amp^r^, pET21a(+) derivative, inducible expression of 6xHis-tagged NsUGT gene at *BamHI/SalI site*		This study
pET21a(+)_SpUGT	Amp^r^, pET21a(+) derivative, inducible expression of 6xHis-tagged SpUGT gene at *BamHI/SalI site*		This study
pMP11	Amp^r^, pKD46 with constitutively expressed Cas9, aTc gRNA targeting ColE1 ori		[41]
pgRNA	Cm^r^, constitutively expressed sgRNA targeting		[41]
pgRNA_pfkA	Cm^r^, constitutively expressed sgRNA targeting pfkA, ColE1 ori		This study
pgRNA_ushA	Cm^r^, constitutively expressed sgRNA targeting ushA, ColE1 ori		This study
pgRNA_atpI	Cm^r^, constitutively expressed sgRNA targeting atpI, ColE1 ori		This study
pgRNA_ldhA	Cm^r^, constitutively expressed sgRNA targeting ldhA, ColE1 ori		This study

^*^Abbreviations: Amp: ampicillin; Cm: Chloramphenicol; Km: kanamycin; and r: resistance

### Mining of crocetin dialdehyde dehydrogenase (crALDH)

Putative crocetin dialdehyde dehydrogenases (crALDH) were investigated by using the blastp program with default parameters against the NCBI non-redundant protein databases, limited to *Synechocystis* strains and *C. sativus*. AldH6803 (GenBank accession number WP_010873792.1) from *Synechocystis* sp. PCC6803 and CsALDH3I1 (GenBank accession number AWN56749.1) from *C. sativus* were used as query amino acid sequences. The blastp program output yielded six amino acid sequences of candidate ALDHs: ALDH7942 (GenBank accession number AAB33154.1), CsALDH2B4 (AWO14302.1), CsALDH2C4 (AWN56744.1), CsALDH5F1 (AWN56745.1), CsALDH6B2 (AQM36715.2), and CsALDH7B4 (AWN56746.1). Evolutionary relationships of the eight ALDHs (including AldH6803 and CsALDH3I1) were inferred using the neighbor-joining method [42], and phylogenetic analysis was performed using the MEGA-X program [43].

### Mining novel glycosyltransferases (UGT) proteins

Putative crocetin glycosyltransferases (UGT-1) were examined by running the blastp program with default parameters against the NCBI non-redundant protein databases using the query amino acid sequence of UGT75L6 (named GjUGT1, GenBank accession number F8WKW0) from *Gardenia jasminoides*. Four amino acid sequences of candidate UGT-1 proteins were mined: NtUGT (GenBank accession number XP_016468459.1), StUGT (XP_006358760.1), FaGT2 (Q66PF4) and GT1-316 (A0A076GGW6). Similarly, potential crocin-2 glycosyltransferases (UGT-2) were investigated using the query amino acid sequence of UGT94E5 (named GjUGT9, GenBank accession number F8WKW8) from *G. jasminoides*. Three putative UGT-2 amino acid sequences were retrieved: SpUGT(GenBank accession number XP_015057190.1), NsUGT (XP_009793442.1), and CaUGT3 (C5NN14). We used the neighbor-joining method to infer the evolutionary relationships of the nine UGTs (including GjUGT1 and GjUGT9) and constructed a phylogenetic tree using the MEGA-X program [43].

### Cloning of pathway genes and construction of expression plasmids for pathway genes

All plasmids and primers used in this study are listed in Table 2 and Additional file 1: Table S1. The pathway genes were cloned, and expression plasmids for the pathway genes were constructed using conventional restriction enzyme-based cloning and uracil excision cloning technology (USER). Using gene-specific primers, two *aldH* genes were amplified by PCR from genomic DNA (gDNA) of *Synechocystis* sp. PCC 6803 and *Synechocystis elongatus* PCC 7942. Each PCR product was digested and ligated into plasmid pUCM (Table 2), creating pUCM_A6803 and pUCM_A7942 and subcloning the *aldH* gene from pUCM_A6803 and pUCM_A7942 into plasmid pBBR1MCS-2 generated pBBR_A6803 and pBBR_A7942. The *E. coli* codon-optimized versions of the plant-derived genes (csCCD2 from *Crocus sativus*, GjUGT1 from *Gardenia jasminoides*, GT1-316 from *Populus fremontii x Populus angustifolia*, NtUGT from *Nicotiana tabacum*, FaGT2 from *Fragaria ananassa*, StUGT from *Solanum tuberosum*, CaUGT3 from *Catharanthus roseus*, NsUGT from *Nicotiana sylvestris*, and SpUGT from *Solanum pennellii*) were chemically synthesized (GenScript, Piscataway, NJ, USA) and individually subcloned into a plasmid pKK223-3 (Table 2; Additional file 1: Table S2 for a sequence of the synthetic genes). The cloned pathway genes were modularly assembled into the plasmid pSTVM via USER technology, yielding pSTVM_CsCCD2-A6803(M), pSTVM_CsCCD2-A7942(M), pSTVM_CsCCD2-A6803(P), and pSTVM_CsCCD2-A7942(P) (Table 2).

### Integration of zeaxanthin-biosynthetic genes into *E. Coli* chromosome

Utilizing the gDNA of *Pantoea agglomerans*, PCR was used to amplify five zeaxanthin biosynthetic genes (*crtE*, *crtB*, *crtI*, *crtY*, and *crtZ*). The zeaxanthin pathway was constructed using the isoprenyl pyrophosphate (IPP)-overproducing *E. coli* MGI strain [39], which overexpresses *dxs*, *dxr*, *idi*, and *ispA* on the *E. coli* genome, as a platform strain. First, a synthetic expression module consisting of a constitutive promoter, *crtE* gene, and terminator was integrated into the *pfkA* [44] and *ushA* sites [45] in the MGI genome, yielding the MGI2E strain containing two copies of the *crtE* gene. A synthetic module expressing c*rtY, crtI*, and *crtB* was then inserted into the *atpI* site [35] of the MGI2E genome, resulting in MGI2EBIY. Finally, a synthetic module expressing *crtY* and *crtZ* was integrated into the *ldhA* site [46] of the MGI2EBIY genome, generating MGI2EBI2YZ (named ZEA-1 strain). The genome editing was performed using the CRISPR/Cas9 technique. Using gene-specific primers and an overlapping extension PCR, a linear donor DNA fragment with a combined 250 bp homologous arm sequence specific to the target genome site was constructed (Additional file 1: Table S1). The pgRNA plasmid backbone was amplified by PCR using primers that contained a 20 bp target-specific gRNA sequence to create guide-RNA (gRNA) plasmids. The gRNA sequence was designed using the CHOPCHOP program (https://chopchop.cbu.uib.no). The sequences of the edited genome sites were verified by Sanger sequencing (Macrogen, Seoul, South Korea).

### Expression analysis and in vitro activity assay of UGTs

Each gene encoding a UGT candidate on pKK223-3 (Table 2) was subcloned into a IPTG (isopropyl β-D-1-thiogalactopyranoside) inducible plasmid pET21a (+) vector with gene-specific primers, constructing pET21a(+)_GjUGT1, pET21a(+)_GT1-316, pET21a(+)_NtUGT, pET21a(+)_FaGT2, pET21a(+)_StUGT, pET21a(+)_GjUGT9, pET21a(+)_CaUGT3, pET21a(+)_NsUGT, and pET21a(+)_SpUGT. The UGT candidate-containing pET21α(+) plasmids were transformed into *E.coli* BL21 strain, and the recombinant strain was cultured at 30 ℃ to an OD_600_ of 0.6 before being supplemented with 1 mM IPTG to induce the expression of UGTs. Cells cultivated for 3 h after induction were harvested and washed with 50 mM Tris-HCl (pH 7.0) buffer. Harvested cells were lysed at 4 ℃ in 10 mL of 50 mM Tris-HCl (pH 7.0) containing 0.2 mg/mL lysozyme and 0.1 mM PMSF using an ultrasonicator (Branson, USA). SDS-PAGE analyzed the expressions of UGT candidates. An in vitro activity assay was performed on six UGT candidates (GjUGT1, NtUGT, GT1-316, SpUGT, NsUGT, and CaUGT3), whose expression in crude protein extracts from *E. coli* was confirmed. Protein concentration was measured using a Bradford assay kit (Sigma-Aldrich, St. Louis, MO, USA). The previously described procedure was to be used with a slight modification for the in vitro assay of crocetin glycosyltransferase activity of UGT-1 candidates [31]. Briefly, 25 µg of the crude protein extract of the UGT-1 candidate was added to a reaction mixture [50 mM Tris-HCl (pH 7.0), 5 mM UDP-glucose, 0.1 mM crocetin encapsulated with maltosyl-β-cyclodextrin]. After 2 h of incubation at 30 ℃, an equal volume of chilled methanol (100 µL) was added to the reaction mixture, which was then centrifuged for 5 min at 4 ℃ and 13,200 rpm. For the analysis of crocin-1 or crocin-2, the reaction mixture was analysed by Agilent 1260 high-performance liquid chromatography (HPLC; Agilent Technology, Santa Clara, CA, USA) equipped with a photodiode array detector and a ZORBAX Eclipse XDB-C18 column (150 mm × 4.6 mm, 5 μm, Agilent Technology) at 40 °C was then used to analyse 10 µL of the filtered supernatant. Elution was performed in a gradient mode at 0.8 mL/min with mobile phase A (100% MeOH) and mobile phase B (100% DDW): mobile phase A: 50–80% (0–60 min), 80–100% (60–80 min), 100 − 50% (80–90 min), and 50% (90–100 min).

No commercial crocin-1 or crocin-2 was available for the in vitro assay of crocin-2 glycosyltransferase activity of UGT-2 candidates. Therefore, crocin-1 and crocin-2 were prepared from 40 µL of the in vitro reaction [50 mM Tris-HCl (pH 7.0), 5 mM UDP-glucose, 0.1 mM crocetin encapsulated with maltosyl-β-cyclodextrin, and 16 µg of crude extract of GjUGT1 or NtUGT] at 30 ℃ for 2 h. Subsequently, 60 µL of 50 mM Tris-HCl (pH 7.0) buffer containing 55 µg of crude UGT-2 candidate and 7.5 mM UDP-glucose were added to the reaction mixture (40 µL), and the 100 µL mixture was incubated for 2 h at 30 ℃. The reaction was terminated by adding 100 µL of chilled methanol. For the analysis of crocin-3 or crocin-4, filtrate from the reaction mixture (10 µL) was analysed using an Agilent 1260 HPLC with mobile phases A (100% acetonitrile) and B (100% DDW) in a gradient elution: mobile phase A: 20–40% (0–20 min), 40–100% (20–25 min), 100% (25-27.5 min), 100 − 20% (27.5–35 min), and 20% (35–45 min).

### Flask and batch bioreactor fermentations

The overnight seed culture was transferred to 500 mL incubating flasks containing 100 mL of TB medium (Tryptone 12 g/L, Yeast extract 24 g/L, 0.17 M KH_2_PO_4_, 0.72 M K_2_HPO_4_, and 10 g/L glycerol) supplemented with the necessary antibiotics for flask fermentation. The flask cultures were grown at 20 ℃ and 250 rpm (except when investigating the effect of culture temperature: The temperature conditions were 30 ℃, 37 ℃, and a shift from 30 ℃ to 20 ℃, with the temperature change occurring at OD_600_ 2.0 in flask fermentation and at OD_600_ 5.0 in bioreactor fermentation). For batch bioreactor fermentation, 150 mL of preculture (with an OD_600_ of 2–3) was inoculated into a 5 L BioFlo 320 bioreactor (Eppendorf, Hamburg, Germany) containing 1.5 L TB medium (Tryptone 12 g/L, Yeast extract 24 g/L, 0.17 M KH_2_PO_4_, 0.72 M K_2_HPO_4_, and 20 g/L glycerol). Batch bioreactor fermentation was carried out at 20 ℃ (or, if necessary, at 30 ℃, 37 ℃ and a shifting mode of 30 ℃ to 20 ℃), pH 7.0, and a dissolved oxygen (DO) level of > 30%. The DO level was maintained by increasing the agitation rate from 300 to 500 rpm and supplying air at 1.0 gas volume per unit medium volume per minute (vvm). The pH was automatically controlled at 7.0 by adding 28% (v/v) NH_4_OH or 2 N HCl solutions. A SpectraMax Plus384 spectrophotometer was used to measure the OD_600_ and thus monitor cell growth. The glycerol concentration was determined using an Agilent 1200 HPLC with an Agilent 1200 refractive index detector, an Aminex HPX-87 H column (7.8 × 300 nm, Bio-Rad, Hercules, CA, USA), and an isocratic mobile phase of 4 mM H_2_SO_4_ at a flow rate of 0.7 mL/min and a column temperature of 50 ℃.

### Extraction and quantification of crocin pathway products

The cell pellets (~ 1 mg wet-cell weight) were repeatedly mixed with 500 µL of a combination of MeOH and acetone (50:50, v/v) until colorless to extract zeaxanthin and crocetin dialdehyde. Crocetin and crocin-1 were extracted using a pH-adjusted MeOH and acetone (50:50, v/v, pH 2.0) mixture as an extraction solvent. After two-phase extraction, the organic solvent phase was filtered through a 0.2 μm PTFE syringe membrane filter and completely dried using a GeneVac EZ-2 centrifugal evaporator (Fisher Scientific, Loughborough, United Kingdom). The dried pellet was dissolved in 100 µL of a 7:1 v/v solution of MeOH and dimethylformamide, and 10 µL of the dissolved sample was analyzed using an Agilent 1260 HPLC as previously described. Crocin-2, 3, and 4 in the culture broth were immediately analyzed using the Agilent 1260 HPLC after filtering with a 0.2 μm PTFE syringe membrane filter. The mass fragmentation spectra of crocin pathway products were monitored using an Agilent LC-Mass 6150 Quadrupole system (Agilent Technologies, Santa Clara, CA, USA) in positive and negative ion modes. The LC-Mass was used to ionize zeaxanthin, crocetin dialdehyde, crocetin, and crocin-1 using an atmospheric pressure chemical ionization source (Agilent Technologies) with the following parameters: 350 °C drying gas temperature, 12.0 L/min drying gas flow, 35 psig nebulizing pressure, 350 °C vaporizer temperature, and 15 µA corona current. Crocin-2, crocin-3, and crocin-4 were ionized using an electrospray ionization source (Agilent Technologies) with the following parameters: 250 °C drying gas temperature, 12.0 L/min drying gas flow, 35 psig nebulizing pressure, 350 °C vaporizer temperature, and capillary voltage set to 2 kV.

Crocin pathway products were identified for structural clarification using a combination of HPLC retention times, UV/Vis spectra, and mass fragmentation spectra in comparison with the authentic standard, if available. For quantification, the authentic standards [zeaxanthin (Sigma-Aldrich, St. Louis, MO, USA), crocetin dialdehyde (Santa Cruz Biotechnology, Dallas, TX, USA), crocetin (MP Biomedicals, Singapore), crocin-3 and crocin-4 (Biopurify Phytochemicals, Chengdu, China) were obtained and used as references. Because crocin-1 and crocin-2 were not commercially available, crocin-4 was used as an alternative reference [19].

### Transcriptional analysis (qRT-PCR)

Recombinant *E. coli* cells (approximately OD_600_) were extracted from a bioreactor and resuspended in RNAlater™ stabilization solution (Invitrogen, Waltham, MA, USA) for quantitative reverse-transcription polymerase chain reaction (qRT-PCR) analysis. The easy-BLUE™ Total RNA Extraction Kit (iNtRON Biotechnology, Seoul, South Korea) was used to extract total RNA. cDNA was synthesized from total RNA samples using the ReverTra Ace™ qPCR RT kit (Toyobo, Osaka, Japan). qRT-PCR was conducted using Rotor-Gene (Qiagen, Hilden, Germany) with a SensiFAST™ SYBR No-ROX One-Step Kit (Bioline, Memphis, TN, USA), and quantification was performed using the comparative Ct (ΔΔCt) method. As a reference gene, the *cysG* gene encoding siroheme synthase was used. The qRT-PCR settings were as follows: 95 ℃ for 2 min, 40 cycles of 95 ℃ for 5s, 60 ℃ for 10 s, and 72 ℃ for 5 s. The primers used for qRT-PCR analysis are listed in Additional file 1: Table S1.

### Electronic supplementary material

Below is the link to the electronic supplementary material.

Supplementary Material 1

## Data Availability

All data generated or analyzed during this study are included in this published article and its Additional file 1.
