# Supplementary material for: Complete microbial synthesis of crocetin and crocins from glycerol in Escherichia coli
Source: Microb Cell Fact. 2024 Jan 4;23:10. doi: 10.1186/s12934-023-02287-9 (PMC10765794; doi:10.1186/s12934-023-02287-9)
Supplement: Supplementary file 1 — Supplementary Material 1 [file 12934_2023_2287_MOESM1_ESM.pdf]

Complete Microbial Synthesis of Crocetin and Crocins from Glycerol in *Escherichia coli*

Jun Ho Lee[1,+], Seong-Rae Lee[1,+] , Sang Yup Lee[2], and Pyung Cheon Lee[1,*]

Table S1. Primers and oligonucleotide used in this study

Table S2. The sequence of *E. coli*-codon optimized genes

Figure S1. Schematical description of zeaxanthin-producing ZEA-1 strain and effect of culture temperature on growth and zeaxanthin production of ZEA-1 strain

Figure S2. Phylogenetic relationships of crALDHs of *C. sativus* (Cs) and *Synechocystis* spp (Syn), inferred using the neighbor-joining method

Figure S3. Phylogenetic tree of UGTs from various organisms

Table S1. Primers and oligonucleotide used in this study

Primer for cloning	Sequence (5'-3')	Final plasmid
pTrc_CrtY_EcoRI_F	cgGAATTCaaggagatataccatgccgcggtatgatctga	pTrc99A_CrtYIB
pTrc_CrtB_SalI_R	acgcGTCGACcgctgcgagaacgtcatg	
pKK_CsCCD2_EcoRI_F	cgGAATTCatggcgaacaaagaagagg	pKK_CsCCD2
pKK_CsCCD2_HindIII_R	cccAAGCTTttaggtctccgcttgatgc	
ald_Syn6803_XbaI_F	gcTCTAGAaggaggattacaaaatgaatactgctaaaactgttgt	pUCM_Syn-aldH6803
ald_Syn6803_EcoRI_R	cgGAATTCctaggagaacaactttttgatta	
aldH_7942_XbaI_F	gctCTAGAaggaggattacaaaatgactgctgtcgttctcc	pUCM_Syn-aldH7942
aldH_7942_EcoRI_R	cgGAATTCctagagcttgcggaagag	
pBBR-pUC-sub-USER-3-F	agacagUctcgaggtcgacggtat	pBBRM_Syn-aldH6803
pBBR-pUC-sub-USER-1-R	atgcaacUagccatcacaaacggcat	
pSTVM2-pUC-sub-USER-1-F	agttgcaUcccgactggaaagcg	
pSTVM2-pUC-sub-USER-3-R	actgtcUatgcggtgtgaaataccg	
sub-SalI_F	acgcGTCGACccgactggaaagcg	pBBRM_Syn-aldH7942
aldH_7942_EcoRI_R	cgGAATTCctagagcttgcggaagag	
pSTVM2-pUC-sub-USER-1-R	atgcaacUcgtaggacag	pSTVM_CsCCD2-Syn-aldH6803(M),
pSTVM2-pUC-sub-USER-3-F	agacagUcataagtgcgg	
pSTVM2-pUC-sub-USER-1-F	agttgcaUcccgactggaaagcg	pSTVM_CsCCD2-Syn-aldH7942(M)
pSTVM2-pUC-sub-USER-3-R	actgtcUatgcggtgtgaaataccg	
ccd2_backbone_F	aatcagcUagcttctgttttggcggatga	pSTVM_CsCCD2-Syn-aldH6803(P),
ccd2_backbone_R	acgctctUaggtctccgcttgatgct	
aldH_F	aagagcgUctagaaggaggattacaaaa	pSTVM_CsCCD2-Syn-aldH7942(P)
aldH_R	agctgatUgtactgagagtgcaccat	
pET_GjUGT1_EcoRI_F	cgGAATTCatggttcagcagcgtcac	pET21a(+)_GjUGT1
pET_GjUGT1_XhoI_R	ccgCTCGAGgttgctctccgcttgatag	
pET_GT1-316_BamHI_F	cgGGATCCatggtctccgagagtctag	pET21a(+)_GT1-316
pET_GT1-316_SalI_R	acgcGTCGACtgcggatgatccgactaac	
pET_NtUGT_BamHI_F	cgGGATCCatggttcaacctcatgtcct	pET21a(+)_NtUGT
pET_NtUGT_HindIII_R	cccAAGCTTacaaccttttcctacttcttgc	
pET_FaGT2_BamHI_F	cgGGATCCatgggtagtgagtctt	pET21a(+)_FaGT2

pET_FaGT2_HindIII_R	cccAAGCTTtgactctactaactcgactt	
pET_StUGT_EcoRI_F	ccgGAATTCatggttcaacctcatgtgctt	pET21a(+)_StUGT
pET_StUGT_XhoI_R	ccgCTCGAGacaagattttcccacttcctg	
pET_CaUGT3_BamHI_F	cgGGATCCatggccaccgaacagca	pET21a(+)_CaUGT3
pET_CaUGT3_SalI_R	acgcGTCGACtacgcacaattgcttcagc	
pET_NsUGT_BamHI_F	cgGGATCCatggacacagaacacagca	pET21a(+)_NsUGT
pET_NsUGT_SalI_R	acgcGTCGACcaacatcatgttattacttttgc	
pUCrop_NtUGT_XbaI_F	gcTCTAGAaggaggattacaaaatggttcaacctcatgtcc	pUCrop_NtUGT
pUCrop_NtUGT_XmaI_R	tcccCCCGGGttaacaaccttttcctacttcttg	
Nt_RBS_CaUGT3_XmaI_F	tcccCCCGGGaggaggacagctaaatggccaccgaacagc	pUCrop_ NtUGT_CaUGT3
Nt_CaUGT3_NotI_R	aaggaaaaaaGCGGCCGCttatacgcacaattgcttcagc	

**Primers used for genome editing**	**Sequence (5′-3′)**	**Donor DNA**
pfkA-UP-F	ggatgtaattatccatcagg	Donor DNA CrtE (pfkAΔ::PLac-CrtE)
pfkA-UP-R	cccgctttccagtcgggaaagactacctctgaactttgg	
pLac-CrtE-F	tttcccgactggaaagcg	
pLac-CrtE-R	atgcggtgtgaaataccg	
pfkA-DOWN-F	tgcggtatttcacaccgcatattaccgaacatatgtgtgat	
pfkA-DOWN-R	atcgacgctatcgaaaaca	
ushA-UP-F	ggcagaaaacaaaaaacagg	Donor DNA CrtE (ushAΔ::PLac-CrtE)
ushA -UP-R	cccgctttccagtcgggaaactggttttcagcgatttcag	
pLac-CrtE-F	tttcccgactggaaagcg	
pLac-CrtE-R	atgcggtgtgaaataccg	
ushA -DOWN-F	tgcggtatttcacaccgcattggatgtgagtgtttatgaac	
ushA -DOWN-R	cactggggcagaaaaaacg	
atpI-UP-F	ttgttgtgtaaatgtcattta	Donor DNA CrtYIB (atpIΔ::PTrc-CrtYIB)
atpI-UP-R	tagccaaaaggtgaataaat	
trc_YIB_F	atttattcaccttttggctagtcatcaccgaaacgcgc	
trc_YIB_R	taaggcttagagtcaagcattgtagaaacgcaaaaaggcc	
atpI-DOWN-F	atgcttgactctaagcct	
atpI-DOWN-R	gcaaatatcataaacaaaacg	
ldhA-UP-F	gataacggagatcgggaatg	Donor DNA CrtYZ
ldhA-UP-R	tttgtgctataaacggcgagt	

trc_CrtY_OV_F	ctcgccgtttatagcacaaattagcgcgaattgatctggt	(ldhAΔ::PTrc-CrtYZ)
trc_CrtY_OV_R	tcattgcatcgcctgttga	
CrtZ_OV_F	tcaacaggcgatgcaatgaaaggagatataccatgttgtggatttggaatgc	
CrtZ_OV_R	aggtttcgcctttttccagattacttcccgggtggcg	
ldhA-DOWN-F	tctggaaaaaggcgaaacct	
ldhA-DOWN-R	ctttggctgtcagttcacca	

**Primer for quantitative analysis of gene transcription levels**	**Sequence (5′-3′)**
CysG_RT_F	atcaggccgcgactattcag
CysG_RT_R	gttcagtttatcgcgcaggc
CsCCD2_RT_F	gcgttgactttccgcgtatc
CsCCD2_RT_R	gaagatcgcttcgctaccgt
AldH7942_RT_F	gtctgcctcaacgacacgat
AldH7942_RT_R	gagcttgcggaagaggttga

* Underlined sequences: sequences of an enzyme digestion site *Capital letter ‘U’ indicate uracil base

Table S2. The sequence of *E. coli*-codon optimized genes

Gene	Sequence
CsCCD2	ATGGCGAACAAAGAAGAGGCGGAAAAACGCAAGAAAAAACCGAAGCCGCTGA<br>AGGTGCTGATTACCAAAGTTGACCCGAAACCGCGCAAGGGTATGGCGAGCGTTG<br>CGGTGGATCTGCTGGAAAAAGCGTTCGTTTATCTGCTGAGCGGCAACAGCGCGG<br>CGGATCGTAGCAGCAGCAGCGGTCGTCGTCGTCGTAAAGAGCACTACTATCTGA<br>GCGGTAACTACGCGCCGGTTGGTCACGAAACCCCGCCGAGCGATCACCTGCCGA<br>TCCACGGTAGCCTGCCGGAGTGCCTGAACGGTGTGTTTCTGCGTGTGGGTCCGA<br>ACCCGAAGTTCGCGCCGGTGGCGGGTTACAACTGGGTTGACGGTGACGGCATGA<br>TTCACGGTCTGCGTATCAAAGACGGTAAAGCGACCTATCTGAGCCGTTACATCA<br>AGACCAGCCGTTTCAAGCAAGAGGAATACTTCGGTCGTGCGAAATTCATGAAGA<br>TTGGCGATCTGCGTGGTCTGCTGGGCTTCTTTACCATTCTGATCCTGGTGCTGCG<br>TACCACCCTGAAGGTGATCGATATTAGCTATGGTCGTGGCACCGGTAACACCGC<br>GCTGGTGTATCACAACGGTCTGCTGCTGGCGCTGAGCGAGGAAGACAAGCCGTA<br>TGTTGTGAAAGTTCTGGAGGATGGTGACCTGCAAACCCTGGGTATCCTGGATTA<br>CGACAAGAAACTGAGCCACCCGTTCACCGCGCACCCGAAGATTGACCCGCTGAC<br>CGACGAAATGTTTACCTTCGGCTATAGCATTAGCCCGCCGTATCTGACCTATCGT<br>GTGATTAGCAAAGACGGCGTTATGCAGGACCCGGTTCAGATCAGCATTACCAGC<br>CCGACCATTATGCACGATTTCGCGATTACCGAAAACTATGCGATCTTCATGGATC<br>TGCCGCTGTACTTTCAGCCGGAAGAGATGGTGAAGGGTAAATTCGTGAGCAGCT<br>TCCACCCGACCAAACGTGCGCGTATTGGTGTTCTGCCGCGTTACGCGAAAGATG<br>AACACCCGATTCGTTGGTTTGACCTGCCGAGCTGCTTCATGACCCACAACGCGA<br>ACGCGTGGGAGGAAAACGACGAGGTTGTGCTGTTCACCTGCCGTCTGGAGAGCC<br>CGGATCTGGACATGCTGAGCGGCCCGGCGGAAGAAGAGATTGGCAACAGCAAG<br>AGCGAGCTGTATGAAATGCGTTTCAACCTGAAGACCGGTATCACCAGCCAGAAG<br>CAGCTGAGCGTTCCGAGCGTTGACTTTCCGCGTATCAACCAGAGCTACACCGGT<br>CGTAAGCAACAGTATGTGTACTGCACCCTGGGTAACACCAAGATTAAAGGCATC<br>GTGAAGTTCGATCTGCAAATTGAGCCGGAAGCGGGTAAAACCATGCTGGAAGTT<br>GGTGGCAACGTTCAAGGCATTTTTGAACTGGGTCCGCGTCGTTACGGTAGCGAA<br>GCGATCTTCGTTCCGTGCCAACCGGGTATTAAAAGCGATGAAGACGATGGTTAC<br>CTGATTTTCTTTGTGCACGACGAAAACAACGGTAAAAGCGAAGTTAACGTGATT<br>GATGCGAAAACCATGAGCGCGGAGCCGGTTGCGGTGGTTGAGCTGCCGAGCCG<br>TGTGCCGTATGGTTTCCACGCGCTGTTCCTGAATGAAGAAGAGCTGCAAAAGCA<br>TCAAGCGGAGACCTAA
NtUGT	ATGGTTCAACCTCATGTCCTACTTGTTACATTTCCAGCACAAGGACATATTAACC<br>CCTGCTTGCAGTTTGCCATGAGGCTGATCCGTATGGGAATCGAAGTAACGTTTG<br>CTACCTCAGTGTTTGCTCACCGTAGAATGGCTAAGACCGCAACAAGCACCCTGC<br>CCAAGGGCCTAAACTTTGCCGCGTTCAGCGACGGATACGACGATGGGTTTAAGG<br>CAGACGAGCATGATTCCCAGCACTACATGTCTGAAATCAAGTCTAGGGGGAGCG<br>AGACGCTGAAGGATATAATTCTGAAGAGTTCTGATGAAGGGAGACCCGTCACGT<br>CCCTTGTGTATAGCCTGCTTTTACCCTGGGCTGCAAATGTTGCGAGGGAATTTCA<br>CATACCCTGTGCGTTGCTATGGATACAACCGGCAACTGTTTTGGATATTTATTAC<br>TACTACTTTAATGGGAGTGAGGACGCCATAAAAGGATCTACCAACGATCCGAAT<br>TGGTGCATCCAGTTGCCAAATCTACCTCTATTGAAATCTCAAGACTTACCTTCCT<br>TCCTACTGTCCTCAAATAACGATGAGAAATACAGTTTTGCTTTGCCAACGTTCAA<br>AGAACAGCTGGACACATTGGATGTGGAAGAAAACCCCAAGGTTTTGGTCAATAC<br>ATTCGATGCGCTAGAGCAGGAAGAGCTGAAGGCGATTGAGAGATATAATTTAAT<br>CGGGATCGGACCTTTGATACCGAGCAGTTTCTTAGATGGAAAGGACCCGCTGGA<br>TTCAAGTTTTGGTGGCGACTTATTTCAGAAGTCCAACGATTATATTGAATGGCTA<br>AACTCCAAAGATAACAGTAGCGTAATATATATCTCATTCGGTTCACTGTTGAAC<br>CTATCCAAAAACCAGAAGGAGGAAATAGCGAAAGGGCTAATCGAGATAAAAAG<br>GCCATTCTTGTGGGTCATCAGAGACCAGGAAAATGGCAAAGGCGATGAAAAAG<br>AAGAAGAAAAGTTGAGTTGTATGATGGAACTAGAGAAGCAAGGTAAAATCGTA<br>CCATGGTGCTCACAACTAGAAGTCTTGACTCACCCCTCCCTTGGTTGCTTCGTCT<br>CTCACTGCGGTTGGAATTCAACACTTGAGAGTTTGTCTACAGGCGTGCCAGTGG<br>TTGCGTTCCCTCATTGGACCGATCAGGGAACTAACGCGAAACTTATCGAAGACG

	TATGGAAGATCGGTGTGCGTTTGAAGAAAAATGAAGATGGAGTAGTTGAGAGC<br>GAGGAGATAAAAAGGTGTATAGACATGGTTATGAACGGAGGTGAAAAGGGAGA<br>AGAGATGCGTCGTAACGCACAGAAGTGGAAAGAACTAGCTCGTGAAGCCGTTA<br>AAGAGGGAGGAAGTTCTTATATGAACTTAAAAGCCTTCGTGCAAGAAGTAGGA<br>AAAGGTTGTTAA
StUGT	ATGGTTCAACCTCATGTGCTTCTTGTAACGTTCCCGGCACAAGGTCACATCAACC<br>CCTCACTTCAGTTTGCCAAGAGATTAATTAAGATGGGCATCGAGGTTACCTTTAC<br>TACAAGCGTCTTCGCCCACAGACGTATGGCGAAGACGGCTGCTTCCAACGCTCC<br>CAAGGGCCTTAATCTGGCCGCCTTCTCAGATGGTTTGATGATGGCTTCAAATCT<br>AATGTCGATGATTCAAAGCGTTATATGTCTGAGATTAGATCTAGGGGCAGCCAG<br>ACCCTGCGTGACATTATCTTAAAGAGTAGCGACGAAGGAAGGCCCGTAACAAGT<br>CTAGTATATACCCTACTATTGCCTTGGGCTGCTGAAGTAGCAAGGGAGCTGCAT<br>ATTCCTTCAGCTTTGTTATGGATTCAGCCAGCGACAGTCCTAGACATATATTATT<br>ATTACTTCAATGGCTACGAAGATGAGATGAAATGTAGCTCAAACGATCCTAATT<br>GGTCAATACAATTACCTCGTTTGCCCTTACTTAAGTCCCAAGATTTACCCTCTTT<br>CCTTGTGTCCTCTAGCTCCAAGGATGATAAATACAGCTTTGCTCTACCAACGTTC<br>AAAGAACAACTTGACACTCTTGACGGAGAGGAGAATCCCAAGGTCTTAGTGAAT<br>ACGTTTGACGCCCTAGAATTAGAGCCGTTAAAAGCAATCGAGAAGTATAATCTT<br>ATCGGGATTGGACCTCTAATTCCGTCTTCATTCCTGGGCGGGAAAGATTCACTAG<br>AAAGTAGCTTCGGAGGCGATTTATTTCAGAAAAGCGACGATGATTACATGGAAT<br>GGCTAAACACTAAACCCAAATCTTCAATTGTGTACATTTCCTTCGGTTCACTACT<br>TAACCTATCCAGAAATCAAAAAGAGGAAATCGCAAAAGGGTTAATTGAAATCA<br>AGCGTCCTTTTCTGTGGGTCATTAGGGATCAGGAAAATATAAAGGAGGTGGAGA<br>AGGAGGAAGAGAAGTTAAGCTGCATGATGGAGCTGGAAAAACAAGGTAAAATT<br>GTTCCTTGGTGTAGCCAACTAGAGGTATTAACTCATCCCTCCCTGGGATGCTTCG<br>TAAGCCATTGTGGATGGAACAGTACACTTGAAAGTTTATCTTCAGGCGTGCCCG<br>TGGTCGCCTTCCCTCACTGGACAGACCAAGGTACTAACGCGAAATTAATAGAAG<br>ATGTTTGGAAGACAGGTGTACGTATGCGTGTTAGCGAGGATGGCGTCGTCGAAT<br>CAGAGGAGATTAAAAGATGTATAGAGATAGTGATGGATGGGGGCGAGAAAGGC<br>GAGGAAATGAGAAAAAACGCTCAGAAATGGAAAGAACTAGCCAGGGAAGCGG<br>TGAAGGAGGGGGGGAGCAGTGAGGTCAACCTAAAGGCTTTGTGCAGGAAGTG<br>GGAAAATCTTGTTAA
FaGT2	ATGGGTAGTGAGTCTTTGGTTCACGTCTTTCTTGTGTCCTTTATTGGCCAAGGAC<br>ACGTCAACCCGCTTCTGAGGCTGGGGAAAAGACTGGCGGCTAAAGGGCTGCTTG<br>TAACCTTTTGCACGGCGGAGTGTGTCGGGAAAGAAATGAGGAAATCTAATGGG<br>ATAACCGATGAGCCTAAACCAGTAGGAGACGGATTTATACGTTTTGAATTCTTC<br>AAAGATAGGTGGGCAGAAGACGAACCCATGCGTCAGGACCTGGATCTATATTTA<br>CCCCAGTTGGAGCTAGTTGGAAAAGAAGTGATACCTGAGATGATAAAGAAGAA<br>CGCAGAACAAGGGAGACCAGTATCATGCCTAATCAATAATCCGTTTATTCCGTG<br>GGTCTGTGACGTCGCCGAATCTCTTGGTTTACCATCCGCAATGCTGTGGGTTCAA<br>TCTGCCGCCTGTCTAGCTGCTTACTATCACTACTACCATGGACTAGTCCCATTCC<br>CATCTGAGTCTGACATGTTTTGTGATGTCCAAATACCCAGCATGCCATTGTTAAA<br>ATATGACGAGGTTCCCAGCTTTTTGTATCCAACGAGCCCATATCCATTTTTGAGA<br>CGTGCGATCTTGGGTCAATACGGGAACTTAGAGAAGCCGTTTTGTATCCTTATG<br>GATACGTTTCAAGAACTAGAGTCTGAAATAATTGAATATATGGCCAGATTATGT<br>CCCATAAAGGCCGTCGGGCCATTGTTCAAGAATCCCAAGGCTCAAAACGCGGTT<br>AGAGGCGACTTTATGGAAGCCGATGACTCAATCATCGGATGGTTGGACACAAAA<br>CCCAAGAGTTCAGTTGTTTACATTAGCTTCGGGAGCGTTGTATACTTAAAGCAG<br>GAGCAAGTGGATGAAATTGCTCACGGACTTTTATCATCCGGCGTGTCTTTTATAT<br>GGGTGATGAAACCTCCTCATCCGGATAGCGGATTTGAATTGTTGGTATTACCAG<br>AAGGTTTCCTAGAAAAGGCGGGAGACCGTGGAAAAGTTGTGCAATGGAGTCCA<br>CAAGAAAAGATATTAGAACACCCTTCAACTGCATGTTTTGTGACACACTGCGGG<br>TGGAATAGTACGATGGAAAGTTTAACCAGCGGGATGCCCGTGGTCGCGTTTCCA<br>CAGTGGGGGGATCAGGTAACTGACGCGAAGTACCTAGTGGATGAATTTAAGGT<br>AGGAGTTAGGATGTGCAGAGGCGAGGCCGAGGACAGGGTGATTCCTAGAGACG<br>AGGTTGAAAAGTGTCTTTTGGAAGCTACGTCTGGTTCAAAAGCTGCAGAGATGA<br>AACAAAATGCGCTTAAGTGGAAAGCTGCTGCAGAGGCTGCGTTCTCCGAAGGA<br>GGATCTTCAGATAGGAATCTGCAAGCCTTCGTCGACGAAGTCAGACGTATTAGC<br>GCCAGCTTAAATTCTAAGTCCTCTGCTGTCGGCTATGTCAAGAGTAAGATCAAC

	GGAGTGGTGGAATATGTCGATAGTAAGTTAAACGGGAAAGCCGCCCCGGTGGA<br>GGAGGCGAACACTCGTACAAATGGCATCGCAAAGGTAGAACAACCTAAGGCAG<br>CAAACGGCAAGGTGGAAATAGCTGAATTAACTCCGATTAATGGCAAGGTTGAG<br>ATTGCCGAACTGAAGCCAATAAACGGGAAAGTCGAGTTAGTAGAGTCATAA
GT1-316	ATGGTCTCCGAGAGTCTAGGTCACCTATTCCTAGTATCTTTTCCGGGGCAGGGGC<br>ATGTAAACCCTTTATTAAGATTAGGAAAAATCCTTGCTAGTAAAGGTTTCCTAGT<br>TACCTTTTCCACCACCGAGACAACAGGGGAGCAGATGCGTAAGGCAAGTGACAT<br>CATAGATAAACTTACACCATTCGGTGACGGCTTCATTCGTTTTGAATTCATTGCC<br>GACGGTTGGGAGGAGGATGAACCCCGTAGGCAAGACCTTGATCAATATCTACTA<br>CAACTTGAATTAGTGGGCAAGCAGGTAATACCGCAAATGATTAAGAAGAATGC<br>CGAACAGGGTAGACCCGTGTCCTGCCTGATCAACAACCCCTTCATCCCATGGGT<br>CACAGATGTCGCTACGACTCTAGGCCTTCCTTCAGCTATGCTGTGGGTACAAAGT<br>TGTGCTTGTTTTGCGAGCTACTATCACTACTACCATGGCACAGTCCCTTTTCCCG<br>ATGAGGAGCACCCCGAAATTGATGTACAGCTACCGTGGATGCCTTTGCTTAAGT<br>ATGATGAGGTCCCTAGTTATCTTTACCCTACGACGCCGTACCCCTTTTTAAGGCG<br>TGCAATATTGGGTCAGTATAAAAACCTGGACAAGCCGTTTTGCATCTTAATGGA<br>GACTTTTGAAGAGCTTGAACCGGAACTTATCAAGCATATGAGTGAAATTTTTCC<br>AATTAAGGCGGTTGGACCGCTATTCCGTAACACAAAAGCGCCCAAAACAACTGT<br>GCACGGCGACTTCTTAAAAGCAGATGATTGTATAGAATGGTTGGATACCAAACC<br>TCCATCTTCTGTCGTTTACGTATCATTCGGCTCTGTTGTGCAGCTAAAACAGGAC<br>CAATGGAACGAGATAGCTTACGGTTTACTTAACTCTGGGGTTTCCTTTTTATTGG<br>TGATGAAGCCCGCTCATAAAGACGCTGGGCATGACCTTCTAGTACTTCCTGACG<br>GCTTCTTAGAGAAAGCTGGCGACAGAGGGAAGGTGGTCCAGTGGTCACCTCAA<br>GAAAAGGTACTAGGTCACCCGAGCGTAGCCTGCTTTGTAACTCACTGTGGATGG<br>AACAGCACGATGGAAGCTCTGACCTCTGGAATGCCCGTGGTCGCTTTTCCCCAG<br>TGGGGTGATCAGGTTACAAATGCGAAATACTTGGTAGACATACTAAAGGTAGGA<br>GTCAGGATGTGTAGGGGAGAGGCCGAAAATAAGCTTATTACAAGAGATGAAAT<br>TGAAAAATGCCTATTAGAAGCTACGGTAGGACCTAAAGCTGTTGAGATGAAGCA<br>AAACGCCATGAAATGGAAGGAAGCTGCTGAAGCCGCGGTCGCCGAAGGTGGCT<br>CAAGTGACCAAAACATACGTTACTTTACTGATGACATCGTGAAAGCAAATGAGT<br>CCGAGATTGCTAGAAAGTGTATCGGCTCTAACGAATTCCCGGTGTCAGTCGTTG<br>TTAAATCTAATGAGAAAGTGGATGAGTTAGTCGGATCATCCGCATAA
GjUGT1	ATGGTTCAGCAGCGTCACGTTTTGTTGATTACCTATCCAGCACAGGGTCACATTA<br>ACCCAGCGTTGCAGTTCGCACAGAGATTGTTGCGTATGGGTATTCAAGTTACCCT<br>GGCGACCAGCGTGTACGCTCTGAGCCGTATGAAAAAGAGCAGCGGTAGCACCC<br>CGAAAGGTCTGACCTTTGCAACCTTCAGCGATGGTTACGATGACGGTTTTCGTCC<br>GAAAGGTGTTGACCATACCGAATATATGAGCAGCCTGGCGAAGCAAGGTAGCA<br>ATACCCTGCGTAATGTGATCAACACCAGCGCTGATCAGGGTTGCCCGGTTACCT<br>GTTTGGTGTATACCTTGTTGCTGCCATGGGCTGCTACCGTTGCACGTGAATGCCA<br>CATTCCGAGCGCGCTGCTGTGGATCCAACCGGTTGCTGTGATGGATATCTACTAT<br>TACTATTTCCGTGGTTATGAAGATGACGTTAAGAATAACAGCAATGACCCGACC<br>TGGAGCATCCAGTTTCCGGGTCTGCCGAGCATGAAAGCTAAGGATCTGCCGAGC<br>TTCATTCTGCCGAGCAGCGACAACATCTACAGCTTTGCACTGCCGACCTTCAAA<br>AAGCAACTGGAAACCCTGGATGAAGAAGAACGTCCGAAAGTTCTGGTGAATAC<br>CTTTGACGCGCTGGAACCGCAGGCACTGAAGGCGATTGAAAGCTATAACCTGAT<br>TGCAATTGGTCCACTGACCCCGAGCGCGTTCCTGGATGGTAAAGACCCGAGCGA<br>AACCAGCTTTAGCGGTGACCTGTTTCAGAAAAGCAAGGACTACAAGGAATGGCT<br>GAATAGCCGTCCGGCTGGTAGCGTTGTGTATGTTAGCTTTGGTAGCCTGCTGACC<br>CTGCCGAAACAACAGATGGAAGAAATTGCACGTGGTCTGCTGAAGAGCGGTCG<br>TCCGTTCCTGTGGGTTATTCGTGCGAAAGAAAACGGTGAAGAAGAAAAGGAAG<br>AAGATCGTCTGATTTGCATGGAAGAACTGGAAGAACAGGGTATGATTGTTCCGT<br>GGTGTAGCCAGATCGAAGTGCTGACCCATCCGAGCCTGGGTTGCTTTGTTACCC<br>ACTGTGGTTGGAATAGCACCCTGGAAACCCTGGTTTGCGGTGTGCCGGTTGTGG<br>CTTTCCCGCATTGGACCGATCAAGGTACCAATGCAAAACTGATCGAAGACGTTT<br>GGGAAACCGGTGTGCGTGTTGTGCCGAACGAAGATGGTACCGTTGAAAGCGAC<br>GAAATTAAACGTTGTATCGAAACCGTTATGGATGACGGTGAAAAAGGTGTGGA<br>ACTGAAGCGTAACGCGAAAAAGTGGAAGGAACTGGCTCGTGAAGCAATGCAGG<br>AAGATGGTAGCAGCGACAAAAACCTGAAAGCGTTCGTGGAGGATGCGGGCAAG<br>GGctatCAAGCGGAGAGCAACTAA

SpUGT	ATGGGAACTCAAGTTACAGAACATGGAACATCTAATCTAAGGGTAGTCATGTTC<br>CCTTGGCTGGCATACGGTCACATCTCACCATTCCTTTATGTCGCAAAAAAACTGG<br>CAGACAGGGGTTTCCTGATTTACTTATGTAGTACTCCCATTAATCTTAAGTCCAC<br>AATAAAGAAAATACCAGAAAAATACGCCGACTCCATCCATTTAATCGAGCTTCA<br>TATTCCAGAATTGCCAGAGTTGCCTCCTCATTATCATACCACGAACGGCCTTCCA<br>CCTCATCTAAATCACACACTACAGAAGGCCTTGAAGATGTCAAAGCCCAATCTA<br>TCCAAAATACTAAAAAACCTTAAGCCAGATTTAATGATCTACGACGTACTACAA<br>CAGTGGGCTGAGAGGGTAGCGAACGAACAGTCCATTCCGGCTGTAAGGTTATTA<br>ACCTTCGGTGCCGCAGTTTTTTCATATTTTTGCAATTTGGTTAAGAAACCCGGAG<br>TCGAGTTTCCATTCCCCGACATTTATTTGAGAAAAATTGAGCAGGTCAAACTAG<br>GTGAAATGTTGGAGAAATCTGCCAAAGACCAAGATCCTGACGATGAAGAAAGG<br>TTAGTAGATGAGTACAAACAAATTGCTTTAATTTGTACCAGTAGAACTATTGAA<br>GCCAAATACATCGACTTCCTATTGGAGCTGAGTAACCTAAAGGTCGTTCCAGTC<br>GGTCCTCCCGTCCAGGACTTGATCACCAATGATGCCGATGATATGGAATTAATC<br>GACTGGCTTGGCTCAAAGGATGAAAATTCCACCGTTTTCGTAAGTTTCGGGAGT<br>GAGTATTTTTTAAGCAAAGAAGATATGGAAGAGGTCGCGCTAGGCCTTGAACTG<br>AGCAACGTTAACTTTGTATGGGTCGCTAGGTTCCCCAAGGGAGAAGAACAAAAT<br>CTAGAAGATGCCTTACCGAAGGGGTTCCTGGAGAGAATAGGGGAACGTGGGAG<br>AGTCCTGGATAAGTTTGCACCGCAGCTGAGGATATTAAATCACACTTCCACTGG<br>TGGATTTATATCACATTGTGGTTGGAACAGCGTGATGGAATCTATACACTTCGG<br>AGTTCCGATTGTGGCGATGCCAATGCATTTGGACCAGCCAATGAACGCGAGGCT<br>TATCGTTGAGCTTGGAGTAGCGGTGGAGATTGTTAGGGACGATGATGGCAAAAT<br>TCACAGGGAAGAAATTGCGAAGACTCTTAAGGACGTGATAACAGAGCGTATTG<br>GAGAGAATCTAAGGGCTAAGATGCGTGATATTTCAATGAACCTAAACAGTATTA<br>GCGGAGAGGAGATGGACGCAGCTGCTCATGAACTTATACAGTTCTGTAAGATCA<br>ACACCAATTAA
NsUGT	ATGGACACAGAACACAGCACGCTACGTGTGCTAATGTTCCCGTGGCTAGCGCAC<br>GGACACATATCTCCCTATTTAACAGTTGCAAAGAAGCTAGCATGTAGAGGATTT<br>TACGTCTATCTTTGTTCCACGCCGGTCAATCTAAACTTCATCAAGAAGAAGATAC<br>CCCAGAAGTATTCAATTAGTATACAGTTGGTCGAATTCCACCTACCTGACCTACC<br>TGAGCTTCCATTAAGTTACCATACGACAAACGGCATTCCTCCCCACCTAGTGTCA<br>ACGTTAAAAAAAGCCGTAAAGATGAGCAAACCGAATTTTTATAAAATAATTGAG<br>AATTTGAAACCAAATATGTTGATATATGACATTCTTCAGCCATGGGCGAAGGAA<br>GTCGCCAACTCCTACAATATCCCCGCTGTTATGTTATTGACGTTTTGCGCTGCCA<br>TGCTTTCTTACCGTTTGCACCCGGTCAAAAAGCCGGGCACAGAGTTCCCATTCCC<br>GGCGCTATATTTGAGGAAAATTGAACGTCAGCAAAGGGAGGAGATGCTTGAAA<br>AAGCCGCCAAGGAGAAGGACCCGGATGATAAAGACCCGTTTGCGGAGGAAGAA<br>ACTATGAACAAGATAATCCTGATGTCTACAAGCAGGGCGACGGAGGCCAAGTA<br>CATCGACTATTTCACTGAACTAATTCAGTGGAAGATAATTCCTGTGGGCCCGCCC<br>GTCCAGGAAACGACTAACGAGTACGACGGAGATGTAGATGATTTAATTGATTGG<br>TTGGGAAACAAATATGAAAACTCCACTGTATTTGTCAGCTTTGGGAGCCAATAT<br>TTCTTGAGCAAAGAAGACTTAGAGGAGATTGCTCTAGGATTGGAACTATCCAAT<br>GTGAACTTCATATGGGTCGTGAGATTCCCCAAGGGGGAGGAAGTTAGAGTAGA<br>AGAAGCGTTGCCAGAAGGTTTCCTTGAGAGGATTGGGGACAGAGGGAGGGTTG<br>TTGACGGTTGGGCACCTCAATTGCGTATTCTGAGTCACCCTTCAACCGGAGGATT<br>TGTCAGTCACTGTGGGTGGAACTCTGTGATGGAGTCCATCGATTTTCGTGTCCCT<br>ATCATCGCCCTGCCAATGCACCTGGATCAGCCAATCAATGCCCGTCTAATTGTCG<br>AATTAGGAGTTGCCGTCGAAATAGTTCGTGACGACGAGGGCAAAGTTCACAGG<br>GGGGAGGTAGCAGAGATCGTTAAAAGCATTATATGTGAGAAGACGGGGGAAAA<br>TTTGCGTAATAAAGTCCGTGAAATCTCTGAAAATCTTAAGAAAGAAAGGGAAGA<br>GGAGATGGACGCAGCGATAGGAGAACTGGTGCAATTATGCAAGACTAGCAAAA<br>GTAATAACATGATGTTGTAA

CaUGT3	ATGGCCACCGAACAGCAACAGGCATCCATCTCTTGTAAAATTTTGATGTTCCCTT<br>GGCTAGCTTTCGGACATATCTCCAGCTTCTTGCAATTAGCGAAGAAGCTTAGCG<br>ACAGGGGGTTCTATTTTTATATCTGCTCCACCCCTATAAATCTTGACTCCATAAA<br>AAACAAAATAAATCAGAATTATTCATCCTCCATTCAGCTAGTCGATCTGCACCT<br>ACCCAATAGCCCTCAGTTGCCCCCGAGTTTACACACAACCAACGGACTACCGCC<br>GCATTTGATGTCAACTCTGAAGAATGCGCTTATTGACGCTAATCCCGATCTGTGT<br>AAAATAATAGCAAGCATTAAGCCGGATCTGATCATATATGATCTACATCAACCG<br>TGGACAGAGGCGCTAGCCTCACGTCACAACATTCCTGCGGTCAGTTTCTCTACC<br>ATGAATGCTGTATCCTTTGCCTACGTTATGCATATGTTTATGAATCCGGGCATAG<br>AATTTCCATTCAAAGCTATACATCTGTCTGATTTCGAGCAAGCGCGTTTCTTGGA<br>GCAGCTGGAGTCAGCAAAAAACGATGCCTCAGCAAAAGACCCCGAACTACAGG<br>GAAGTAAAGGGTTTTTTAACTCCACCTTTATAGTTAGATCCAGTCGTGAAATCGA<br>GGGGAAATACGTCGATTACTTGAGTGAGATCTTAAAAAGTAAGGTTATACCTGT<br>TTGCCCCGTCATTAGCCTTAACAATAATGATCAGGGACAAGGGAACAAAGACGA<br>GGACGAAATCATTCAATGGTTGGACAAAAAAAGTCATAGAAGCTCCGTGTTTGT<br>TTCATTCGGTAGTGAATACTTCCTTAACATGCAAGAGATTGAAGAGATAGCCAT<br>TGGTCTTGAGCTGTCTAATGTCAACTTCATCTGGGTTTTGAGGTTTCCCAAAGGT<br>GAAGATACGAAAATAGAAGAGGTGCTACCGGAAGGATTTCTAGATAGGGTGAA<br>GACCAAAGGGAGAATAGTGCATGGCTGGGCCCCACAGGCTCGTATTCTGGGTCA<br>TCCATCTATAGGTGGGTTTGTATCCCATTGTGGCTGGAACAGTGTGATGGAAAG<br>TATTCAAATCGGTGTGCCCATCATTGCGATGCCGATGAATTTAGACCAACCTTTC<br>AATGCAAGGCTTGTCGTGGAAATTGGTGTGGGTATTGAGGTCGGCAGGGACGAG<br>AACGGGAAACTTAAAAGGGAACGTATCGGTGAGGTCATTAAGGAAGTTGCAAT<br>CGGAAAGAAGGGGGAGAAGCTTAGAAAAACCGCGAAAGACCTGGGCCAGAAG<br>CTAAGGGACCGTGAGAAACAGGATTTTGATGAGCTTGCAGCGACGCTGAAGCA<br>ATTGTGCGTATAA

Fig. S1

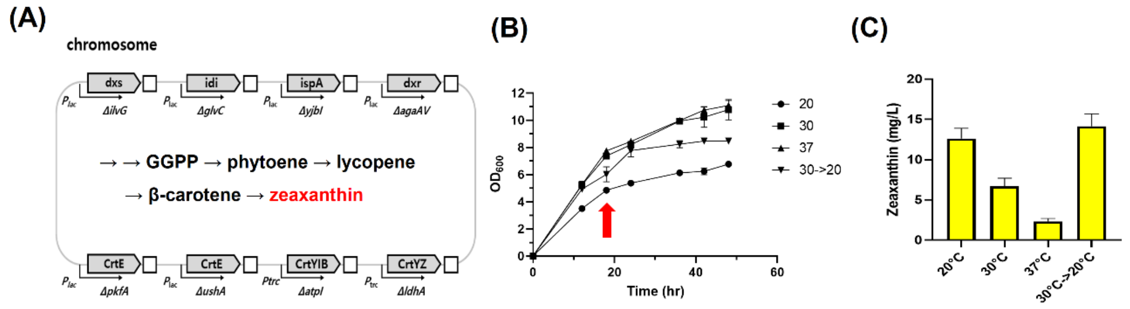

Schematical description of zeaxanthin-producing ZEA-1 strain and effect of culture temperature on growth and zeaxanthin production of ZEA-1 strain. (A) Schematic description of construction of the zeaxanthin-overproducing strain (ZEA-1) by modularly expressing five zeaxanthin biosynthetic pathway genes on the genome of the IPP- overproducing *E. coli* strain. (B) Cell growth of ZEA-1 was monitored at four culture temperatures. An arrow indicates the time of temperature shifting 30 to 20°C. (C) Quantification of zeaxanthin production in the ZEA-1 strains grown at four different temperatures. All experiments were done in biological triplicates and error bars represent mean ± SD.

Fig. S2

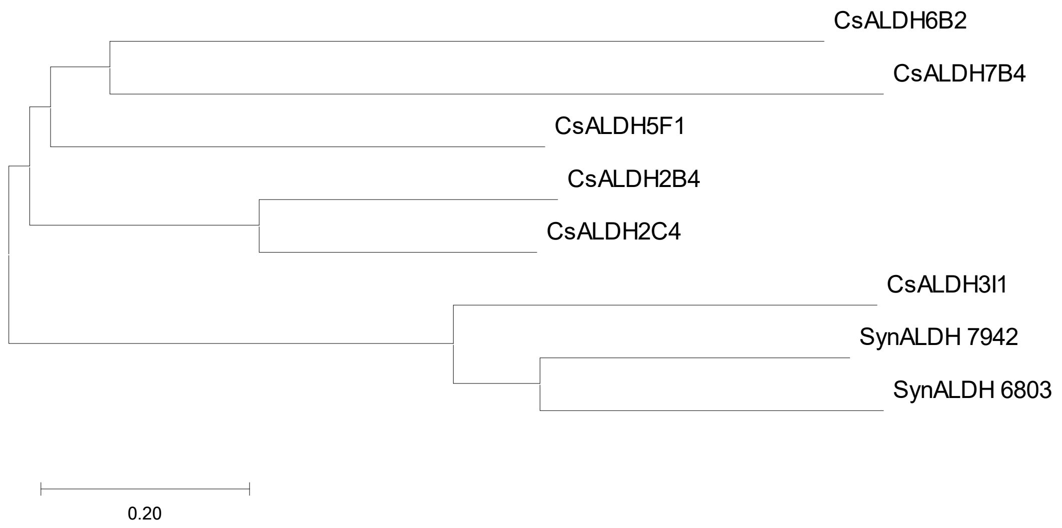

Phylogenetic relationships of crALDHs of *C. sativus* (Cs) and *Synechocystis* spp (Syn), inferred using the neighbor-joining method.

Fig. S3

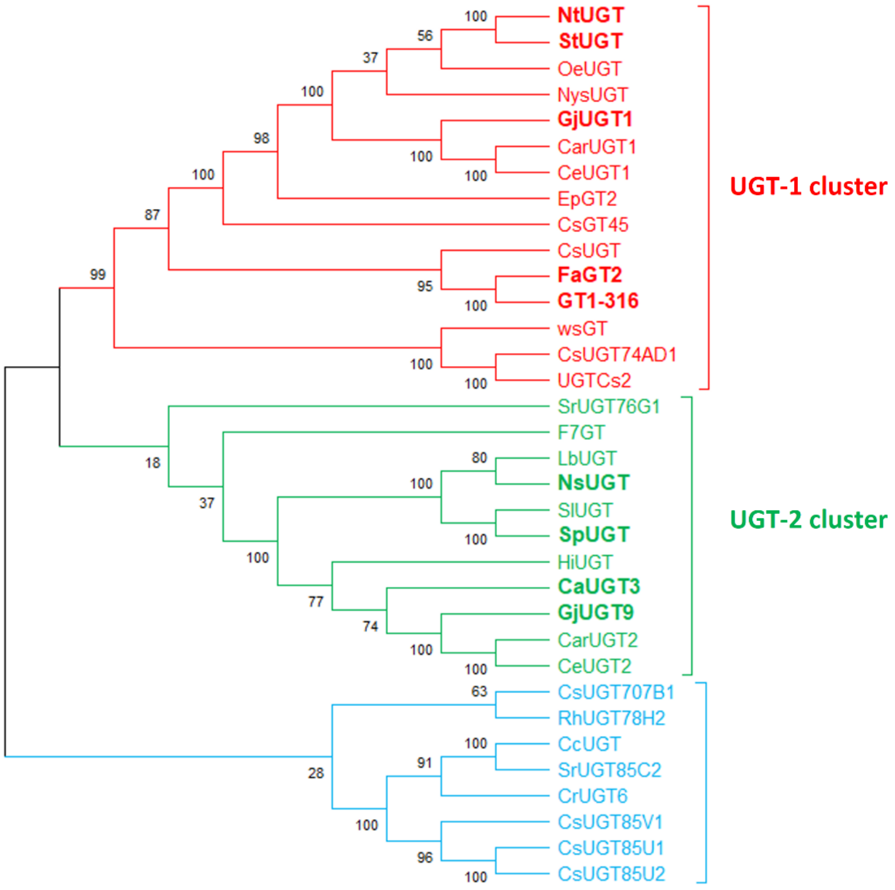

Phylogenetic tree of UGTs from various organisms. The UGTs in red and in green were clustered as first-step UGT-1 (crocetin glycosyltransferase) and second-step UGT-2 (crocin-1/2 glycosyltransferase). Five UGTs in boldface were selected and utilized for the expression and functionality study.
